# New strain *Brevibacillus laterosporus* TSA31-5 produces both brevicidine and brevibacillin, exhibiting distinct antibacterial modes of action against Gram-negative and Gram-positive bacteria

**DOI:** 10.1371/journal.pone.0294474

**Published:** 2024-04-01

**Authors:** Jeongeun Kim, Jueun Kim, Hyosuk Yun, Byambasuren Ganbaatar, Aminallah Tahmasebi, Sun Il Seo, Pyoung Il Kim, Chul Won Lee

**Affiliations:** 1 Department of Chemistry, Chonnam National University, Gwangju, Republic of Korea; 2 Research Center, DAESANG InnoPark, Gangseo-gu, Seoul, Republic of Korea; 3 Department of Agriculture, Minab Higher Education Center, University of Hormozgan, Bandar Abbas, Iran; 4 Center for Industrialization of Agricultural and Livestock Microorganism, Jeongeup-si, Jeollabuk-do, Republic of Korea; Benemérita Universidad Autónoma de Puebla: Benemerita Universidad Autonoma de Puebla, MEXICO

## Abstract

The growing prevalence of antibiotic resistance has made it imperative to search for new antimicrobial compounds derived from natural products. In the present study, *Brevibacillus laterosporus* TSA31-5, isolated from red clay soil, was chosen as the subject for conducting additional antibacterial investigations. The fractions exhibiting the highest antibacterial activity (30% acetonitrile eluent from solid phase extraction) were purified through RP-HPLC. Notably, two compounds (A and B) displayed the most potent antibacterial activity against both *Escherichia coli* and *Staphylococcus aureus*. ESI-MS/MS spectroscopy and NMR analysis confirmed that compound A corresponds to brevicidine and compound B to brevibacillin. Particularly, brevicidine displayed notable antibacterial activity against Gram-negative bacteria, with a minimum inhibitory concentration (MIC) range of 1–8 μg/mL. On the other hand, brevibacillin exhibited robust antimicrobial effectiveness against both Gram-positive bacterial strains (MIC range of 2–4 μg/mL) and Gram-negative bacteria (MIC range of 4–64 μg/mL). Scanning electron microscopy analysis and fluorescence assays uncovered distinctive morphological alterations in bacterial cell membranes induced by brevicidine and brevibacillin. These observations imply distinct mechanisms of antibacterial activity exhibited by the peptides. Brevicidine exhibited no hemolysis or cytotoxicity up to 512 μg/mL, comparable to the negative control. This suggests its promising therapeutic potential in treating infectious diseases. Conversely, brevibacillin demonstrated elevated cytotoxicity in *in vitro* assays. Nonetheless, owing to its noteworthy antimicrobial activity against pathogenic bacteria, brevibacillin could still be explored as a promising antimicrobial agent.

## Introduction

Antibiotics are being used inappropriately in therapeutic, agricultural, and animal husbandry practices, leading to the emergence of drug-resistant strains over the course of several decades [1, 2]. Research has indicated that 95% and 60% of *Staphylococcus aureus* strains exhibit resistance to penicillin and methicillin, respectively [3]. Moreover, resistance genes are capable of being transferred between pathogens, potentially contributing to the escalation of drug resistance [4]. On a global scale, antimicrobial resistance presents a pressing and critical threat to public health. It has been reported that the United States alone witnesses a minimum of 2.8 million antimicrobial-resistant infections each year [5]. Moreover, given the diminishing availability of new antibiotics, the rise in medical expenses, and the heightened mortality rates due to antibiotic resistance, there is a growing inclination towards the development of novel, safe, and efficacious antimicrobial agents [6, 7]. Bacteria are capable of producing a diverse array of highly effective peptide antibiotics [8–10]. Antimicrobial peptides derived from bacteria offer several advantages, such as ease of industrialization, rapid production, and cost-effectiveness. These factors have garnered significant attention and interest [11]. In this context, *Brevibacillus laterosporus* has demonstrated a notable capability for generating a variety of short-sequence peptides [12]. Research has also revealed that the primary structure of antimicrobial peptides isolated from *B*. *laterosporus* plays a pivotal role in influencing their biological activities and physicochemical properties [13]. *B*. *laterosporus* is a Gram-positive bacterium that forms spores and is commonly found in natural habitats such as water, soil, and insects [14]. Indeed, *B*. *laterosporus* has been extensively studied for its substantial potential in various sectors, particularly in agriculture and the food industry [15, 16]. This potential encompasses the ability to inhibit phytopathogens, manage insect populations, and stimulate plant growth, offering diverse benefits in these fields [14, 16–19]. Additionally, this bacterium holds significance as a noteworthy commercial probiotic [20].

Antimicrobial peptides comprise a diverse group of small amino acid molecules that are synthesized across various life kingdoms [21]. These peptides exhibit a broad range of biological functions, including antimicrobial and antitumor activities, wound healing, angiogenesis, and immune regulation, underscoring their evolutionary conservation [22–25]. Antimicrobial peptides function as defensive molecules and hold a pivotal role within the innate immune system of their host organisms [26]. These peptides exert a diverse range of inhibitory effects against bacteria, viruses, parasites, and fungi [2]. Moreover, these peptides exhibit a multitude of structures and functions [27].

Recently, the potent inhibitory activity of brevibacillin, an antimicrobial lipopeptide derived from *B*. *laterosporus*, has been reported against drug-resistant bacteria [28]. Brevicidine is yet another cyclic lipopeptide originating from *B*. *laterosporus*, demonstrating antimicrobial activity primarily against Gram-negative bacteria [29]. Additionally, another analog of brevicidine (brevicidine B) displayed antimicrobial activity against both Gram-positive and Gram-negative bacteria [30].

Promising antimicrobial peptides have garnered significant attention over the past few decades [31]. Antimicrobial peptides are able to prevent the induction of antibiotic-resistant mutants, largely attributed to their rapid physical disruption of bacterial membranes [32]. Furthermore, antimicrobial peptides are regarded as suitable substitutes for antibiotics, given their broad-spectrum and potent activity, high specificity, and low toxicity [27, 33]. The necessity to identify novel bioactive compounds sourced from natural products has grown in significance due to the diminishing efficacy of conventional antibiotics against emerging drug-resistant strains. Moreover, the widespread utilization of antibiotics has led to environmental and agro-ecosystem contamination, resulting in adverse ecological consequences. The presence of antibiotic residues can potentially harm human health and other organisms. Consequently, the consumption of contaminated water and food contributes to the proliferation of resistant bacteria [34–36]. Hence, the primary objective of this study was to explore the isolation, identification, and characterization of antimicrobial peptides obtained from a novel strain of *B*. *laterosporus* TSA31-5, which was isolated from red clay soil. Additionally, to elucidate the antibacterial mechanism of these peptides, both a membrane depolarization assay and scanning electron microscopy (SEM) analysis were carried out. The novel *Brevibacillus laterosporus* strain simultaneously produced brevicidine and brevibacillin peptides that selectively and potently acted on Gram-negative and positive bacteria. These peptides showed excellent antibacterial activities that could address bacterial infections with enhanced antibiotic resistance. These findings will pave the way for further studies on the clinical applications of the antimicrobial peptides.

## Materials and methods

### Isolation and identification of *B*. *laterosporus*

Bacteria with antimicrobial activity were isolated from soil samples collected from Jeonnam Province in Korea (35.034870, 126.730379) and then the bacterial species with the strongest antibacterial activity against Gram-negative *Escherichia coli* and Gram-positive *Staphylococcus aureus*, was selected for further studies. The isolated bacterium was cultured in a tryptic soy agar plate, incubated at 30°C overnight and then a bacterial single colony was transferred to 5 mL of tryptic soy broth and incubated at 30°C overnight with shaking at 180 rpm. After overnight incubation, the optical density at 600 nm of culture broth was adjusted to 1.0, and inoculated with 1 L tryptic soy broth (1%) and incubated at 30°C for 2 d with shaking at 120 rpm. Thereafter, 16S rRNA gene sequencing analysis was conducted to identify the isolated species. Primers for the PCR were 27F (AGAGTGATCMTGGCTCAG) and 1492R (GGTTGTTACGACTT).

### Microbial strains

The following microbial pathogenic strains were used to test the antimicrobial activity of the isolated species and its compounds. A total of six pathogenic bacterial strains [*Acinetobacter baumannii* (ATCC BAA 160f), *Bacillus subtilis* (KCTC 3068), *E*. *coli* (KCTC 1682), *Pseudomonas aeruginosa* (KCTC 1637), *Salmonella typhimurium* (KCTC 1926), and *S*. *aureus* (KCTC 1621)] were purchased from Korean collection for type cultures. Three pathogenic bacterial strains [*Staphylococcus epidermidis* (KCCM 35494), *S*. *epidermidis* (KCCM 40416), and *A*. *baumannii* (KCCM 40203)] were purchased from Korean culture center of microorganisms. A number of five drug-resistant pathogenic bacterial strains [multidrug-resistant *P*. *aeruginosa* (CCARM 2095), multidrug-resistant *P*. *aeruginosa* (CCARM 2109), methicillin-resistant *S*. *aureus* (CCARM 3089), methicillin-resistant *S*. *aureus* (CCARM 3090), and methicillin-resistant *S*. *aureus* (CCARM 3095)] were obtained from culture collection of antimicrobial resistance microbes. Two pathogenic bacterial strains with vancomycin resistance [vancomycin-resistant *Enterococcus faecium* (ATCC 51559) and vancomycin-resistant *Enterococcus faecalis* (ATCC 51575)] and an *E*. *coli* strain which does not synthesize lactose permease, but expresses β-galactosidase [*E*. *coli* (ML 35)] were sourced from American type culture collection.

### Isolation and purification of antimicrobial compounds

The culture broth of *B*. *laterosporus* underwent a two-step purification process to isolate the pure antimicrobial compounds. Initially, the culture broth was subjected to centrifugation at 4000 rpm for a duration of 30 min. Subsequently, the cell-free supernatant was gathered and subjected to filtration using vacuum filtration alongside filter paper, characterized by a diameter of 90 mm and particle retention capability of 30 μm. The obtained supernatant was introduced into C18 solid phase extraction (SPE) cartridges (820 mg sorbent per cartridge, Sep-Pak, Waters). Subsequently, the extract was eluted stepwise using 10 ml of elution solvent containing acetonitrile (ACN) at various concentrations (0%, 10%, 30%, 60%, 90%, 100% by volume). The antibacterial potential of each fraction was evaluated using an agar diffusion assay conducted against both *E*. *coli* and *S*. *aureus*. The elution fractions exhibiting the highest antibacterial activity were singled out for subsequent purification steps. For the second purification phase, a reverse phase-high performance liquid chromatography (RP-HPLC) system (Waters, US) was employed. This process involved utilizing an X-Bridge C18 column with dimensions of 19 × 250 mm and particle size of 5 μm. The mobile phase consisted of solvent A, composed of 5% ACN and 0.05% trifluoroacetic acid (TFA), and solvent B, comprising 95% ACN and 0.05% TFA. The pump B’s gradient program spanned from 25% to 45% over a period of 20 min, while maintaining a flow rate of 10 mL/min. We collected 10 peaks and tested the antibacterial activity of each peak. Subsequently, the purified substances were subjected to lyophilization and then stored at a temperature of -20°C.

### LC-MS analysis

RP-HPLC (Agilent 1100 series) coupled with MS (API 3200 Q TRAP) was employed for the determination of both the molecular mass and purity of the compounds. A X-Bridge C18 column (2.1 × 100mm and a particle size of 3.5 μm) was utilized. The mobile phase was comprised of distilled water (with 0.05% trifluoroacetic acid, TFA, as solvent A) and acetonitrile (with 0.05% TFA, solvent B). Pump B’s gradient program spanned from 5% to 65% over a duration of 30 min. The column oven was maintained at a temperature of 35°C, while the flow rate was set at 0.2 mL/min. The mass range covered by the program was 600–1700 Da in positive mode.

### ESI-MS/MS analysis

To ascertain the amino acid sequences of the purified compounds, ESI-MS/MS analysis was carried out using the API 3200 Q TRAP system. Each compound was dissolved in a solution consisting of 50% ACN containing 0.1% formic acid to achieve a concentration of 100 μg/mL. For the precursor ion, the scan type was set to Q1 MS (Q1), covering a mass range of 500–1600 Da for a duration of 5 min in positive mode. For the subsequent product ion scan, the scan type was configured as product ion (MS2), encompassing a mass range of 100–1600 Da for a duration of 5 minutes, also in positive mode.

### NMR spectroscopy

NMR spectra were acquired using a Bruker Advance 600 MHz spectrometer (Bruker Biospin, Germany) to conduct precise structural analysis. The compounds were dissolved in DMSO-d6 at a concentration of 3 mM to prepare the samples for NMR spectroscopy, which were then analyzed at a temperature of 25°C. Various types of spectra, including ^1^H 2D NMR, double-quantum correlation spectroscopy (DQF-COSY), total correlation spectroscopy (TOCSY), nuclear Overhauser effect spectroscopy (NOESY), heteronuclear single quantum correlation (HSQC), and heteronuclear multiple-bond correlation (HMBC), were acquired and employed for the investigation.

### Antibacterial activity

To validate the antibacterial activity of the compounds, two distinct techniques were employed. Initially, an agar diffusion assay was conducted to ascertain the comprehensive impact of the substances. *E*. *coli* and *S*. *aureus* were cultivated on Luria–Bertani agar plates and incubated at 37°C overnight. A single colony from each bacterium was transferred to 5 mL of Luria–Bertani broth and incubated at 37°C overnight, while being shaken at 180 rpm. Following the overnight incubation, the culture broth was inoculated into 5 mL of Luria–Bertani (LB) broth and subjected to incubation at 37°C with shaking at 180 rpm. When the cell concentration reached an OD_600_ of 0.5, it was measured and then 2-fold diluted using the culture broth. Subsequently, 200 μL of this diluted solution was spread onto a Mueller–Hinton agar plate. The antimicrobial compounds (10 μL) were directly applied to the agar plate, or 20 μL was applied onto a paper disk. The agar plate, containing the pathogenic bacteria along with the compounds, was dried completely and incubated at 37°C for 18 h. The presence of a zone of inhibition was indicative of antibacterial activity. In the second technique, the minimum inhibitory concentrations (MICs) of the compounds were determined using the serial dilution method within a 96-well plate. The pathogenic bacteria were treated in a manner similar to the procedure described for the agar diffusion assay. The culture broth at OD_600_ 0.5 (equivalent to 2 × 10^8^ CFU/mL) was diluted by a factor of 100 using Mueller–Hinton broth (MHB), and 100 μL of this diluted solution was distributed into each well of the 96-well plate. The compounds, dissolved in sterilized water, were diluted with MHB to establish a concentration range of 0.13–256 μg/mL. Subsequently, 100 μL of various concentrations of each compound was mixed with 100 μL of bacterial culture within the 96-well plate. The plate was then incubated at 37°C for 18 h. The MIC was detected as the lowest concentration of peptides that prevented visible turbidity. The MIC was determined by optical density (OD) at 600 nm using a microplate ELISA (Bio-Tek Instruments EL800, San Diego, CA, USA) reader. All experiments were repeated three times.

### Time-dependent killing kinetics assay

The time-dependent killing kinetics of the peptides was assessed using Gram-negative (*E*. *coli*) and Gram-positive (*S*. *aureus*) bacteria. Bacterial cells were cultured in LB media to mid-log phase. The optical density at 600 nm (OD_600_) of cells was adjusted to 0.5 (~10^6^ CFU/mL) in MHB broth. A final inoculum of 10^6^ CFU/mL was treated with 1×MIC of the peptides in 1% peptone. Aliquots were taken at 0 to 180 min and placed on LB agar plate and incubated for 18 h at 37°C. After incubation, the colonies were counted. Each experiment was repeated independently on three separate day.

### Circular dichroism (CD) spectroscopy

Circular dichroism (CD) spectroscopy was employed to validate the secondary structure of the compounds. CD spectra commonly exhibit a negative minimum at wavelengths around 222 nm and 208 nm, alongside a positive maximum at approximately 193 nm when the substance possesses a helical structure. These distinctive signals in the CD spectrum help to characterize and confirm the presence of a helical conformation within the compounds under investigation [37]. The CD spectra were recorded in the range of 250 to 190 nm using a J-810 spectropolarimeter (JASCO, Japan) equipped with a quartz cell with a path length of 1 mm. The compounds were dissolved in different buffers at a concentration of 100 μM. These buffers included 10 mM sodium phosphate buffer (pH 6.8), 30 mM sodium dodecyl sulfate (SDS), 50% (v/v) 2,2,2-trifluoroethanol (TFE), and 0.1% (w/v) lipopolysaccharide of *E*. *coli* in 10 mM sodium phosphate buffer (pH 6.8). The obtained CD signals were then converted to molar ellipticity using the following Eq 1.


$${\left[\theta \right]}_{molar\;ellipticity}\times 10^{-3}(deg\times {cm}^{2}\times {dmol}^{-1})=\frac{\theta_{obs}\times M}{1000\times C\times L}$$ Eq (1)

θ_obs_ is the raw data of the scan value, M is the mean residue molecular weight (molecular weight divided by the number of peptide bonds), C is the protein concentration (mg/mL), and L is the path length of cuvette (mm).

In addition, the percent of α-helix was calculated using the Eq 2 [38].


$$\alpha -helix(\%)=\frac{-100\times (\theta_{222}+3000)}{33000}$$ Eq (2)

θ_222_ is the calculated molar ellipticity at 222 nm.

### Stability test

The molecular stability of brevicidine and brevibacillin was determined based on the relative peak area obtained by RP-HPLC at various pH and heating conditions. Brevicidine and brevibacillin were incubated in pH 2, 7, and 10 tris-based buffers at 25°C for 24 h. For heat stability, the peptides (1mg/mL concentration at pH 7.0) were incubated at 80°C for 120 min. The samples were filtered using the Spin-X Column (0.22 μm, COSTAR, USA). Then, 40 μL of each of the filtered samples was injected into the RP-HPLC system monitored at 230 nm.

### Hemolytic activity assay

The hemolysis assay was conducted to evaluate the cytotoxicity of the pure compounds. Fresh sheep blood was washed three times with 1× phosphate-buffered saline (PBS) and then resuspended in 1× PBS. The suspension of red blood cells (RBCs) was added to a 96-well plate at a volume of 100 μL per well. The pure compounds were dissolved in sterilized water and subsequently diluted with 1× PBS to achieve concentrations ranging from 0.5 to 1024 μL/mL. A volume of 100 μL of the compounds was mixed with 100 μL of prepared RBCs in a 96-well plate, and this process was performed in duplicate for further steps. The plate was then incubated at 37°C for 1 h with gentle shaking at 60 rpm. Following incubation, the plate was centrifuged at 1000 × g for 5 minutes at 4°C. The supernatant from each sample was carefully collected and transferred to another 96-well plate. A microplate reader (PHOmo, China) was employed to measure the absorbance values at 405 nm.

For 100% lysis, Triton X-100 (1%) was used, and 1× PBS served as a blank control. The percentage of hemolysis was calculated using the following equation [39].


$$Hemolysis(\%)=\frac{{OD}_{405nm}(sample)-{OD}_{405nm}(blank)}{{OD}_{405nm}(100\%\;lysis)-{OD}_{405nm}(blank)}$$ Eq (3)

### MTT assay

The mammalian cytotoxicity of compounds was evaluated on RAW264.7 cell using an MTT assay. Dulbecco’s Modified Eagle’s Medium supplemented with 10% fetal bovine serum and 1% antibiotics (penicillin/streptomycin, LONZA, USA) was used as media throughout the experiment. RAW264.7 cells with a concentration of 10^5^ cells/mL were seeded into 96-well plates at 100 μL/well and incubated at 37°C with 5% CO_2_ for 18 h. The plate was then centrifuged at 1000 × *g* for 5 min at 4°C. The media was eliminated using an autoclaved disposable glass Pasteur pipette. A concentration of 1–512 μg/mL of pure compounds was prepared by dissolving them in sterile water and diluting them with media. A volume of 200 μL of each sample was added to 96-well plates and incubated at 37°C with 5% CO_2_. After 24 h of incubation, the media was eliminated in the same way and 100 μL of MTT solution (1000 μg/mL of thiazolyl blue tetrazolium bromide) was added to each well and incubated at 37°C for 4 h with 5% CO_2_. The supernatant was removed in the same way and 100 μL of DMSO was added to each well of the plate and incubated at 37°C for 20 min with 5% CO_2._ Absorbance values were measured at 550 nm using a microplate reader (PHOmo, China). Plain media was used as a negative control, and 100% DMSO served as a blank. The percentage of cell viability was calculated using the Eq 4.


$$Cell\;viability(\%)=\frac{{OD}_{550nm}(sample)-{OD}_{550nm}(blank)}{{OD}_{550nm}(negative\;control)-{OD}_{550nm}(blank)}$$ Eq (4)

### Membrane depolarization assay

To assess the mechanism of action of the antibacterial activity, cytoplasmic membrane depolarization was evaluated using a cyanine dye known as 3,3ʹ-dipropylthiadicarbo cyanine iodide (DiSC3-(5)). *E*. *coli* and *S*. aureus were cultured at 37°C with agitation until reaching an optical density (OD_600_) of 0.4. The bacterial cells were then harvested by centrifuging the culture broth at 3000 rpm for 5 min. Bacterial cells were washed three times with washing buffer and resuspended to the OD_600_ of 0.05 in the similar buffer. The spectrophotometer (HITACHI F-4500 FL) using a quartz cuvette (Hellma, 101.650QG) was used to monitor and record the fluorescence intensity with an excitation wavelength of 620 nm and an emission wavelength of 670 nm. The temperature of the instrument was maintained at 20°C. *S*. *aureus* was resuspended to an OD_600_ of 0.05 with buffer (5 mM HEPES, 20 mM glucose, and 10 mM KCl at pH 7.4). The bacterial suspension was adjusted to an optical density (OD_600_) of 0.05, and then DiSC3-(5) was introduced to achieve a final concentration of 2 μM. This mixture was pre-incubated at 25°C for 1 h. Subsequently, after 100 seconds, the peptide was added at varying concentrations (0.5× MIC, 1× MIC, 2× MIC).

### SEM analysis

Scanning Electron Microscopy (SEM) analysis was conducted to ascertain the effect of the compounds on the bacteria. *E*. *coli* and *S*. *aureus* (adjusted to an OD_600_ of 0.5) were cultured in MHB and diluted to an OD_600_ of 0.1. The bacterial cells were then incubated at 37°C for 4 h with the peptides at the final concentrations of 0.5× MIC, 1× MIC, and 2× MIC. A control treatment with no peptide was included for comparison.

### Statistical analysis

All graphing and statistical analysis were carried out using a one-way analysis of variance (ANOVA) with SPSS 16.0 software and Sigma plot v12.0. The data in this study were expressed as the means ± standard deviation (SD) from three independent experiments.

## Results

### Isolation and identification of *B*. *laterosporus* TSA31-5

**Fig 1:**
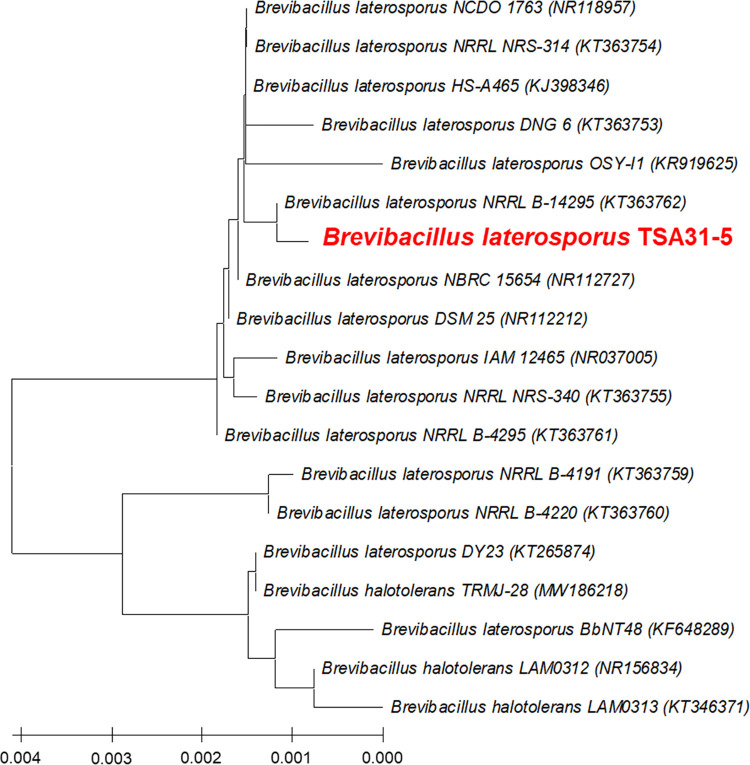
Neighbor-joining phylogenetic tree of the TSA31-5 strain. The tree was generated based on analysis of the 16S rRNA gene sequence using MEGA ver. 11.0.8. The neighbor-joining method was employed for tree construction.

Bacteria with antimicrobial activity were isolated from red clay. We deposited the *B*. *laterosporus* TSA31-5 strain in the Korean Collection for Type Cultures with the accession number KCTC15158BP. The bacterium TSA31-5 strain with the highest antimicrobial activity was selected and identified using 16S rRNA gene sequence analysis. The GenBank accession number for the 16S rRNA of TSA31-5 strain is OR298234. The results confirmed that it is a strain of *B*. *laterosporus* with 99% nucleotide identity (Fig 1).

### Purification of antimicrobial compounds

**Fig 2:**
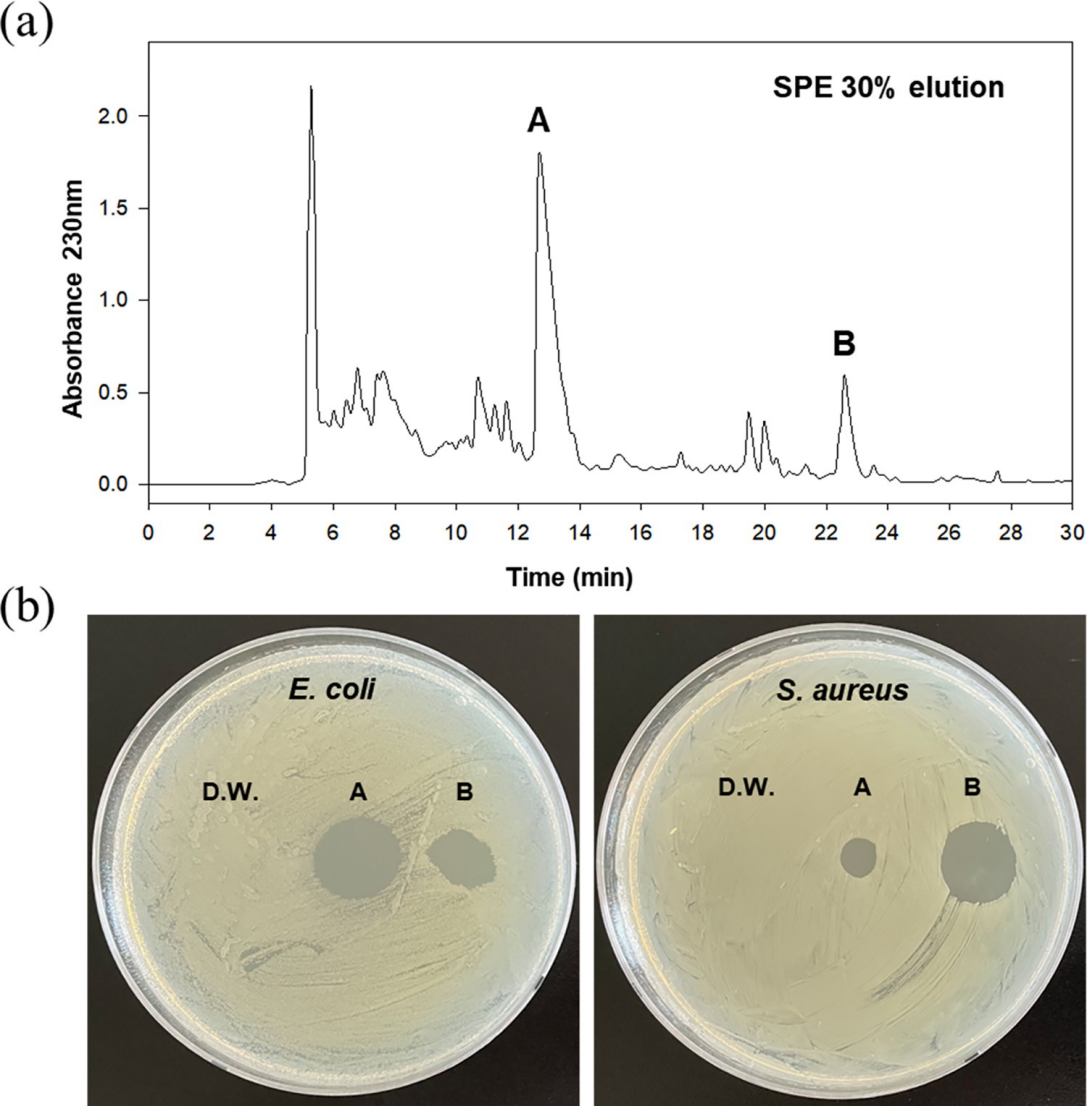
RP-HPLC analysis and antibacterial activity of the 30% acetonitrile eluent obtained from solid phase extraction of TSA31-5 culture media. (a) RP-HPLC chromatogram. (b) Antibacterial activity of compound A and B in the 30% eluent against *E*. *coli* and *S*. *aureus*.

Solid phase extraction using C18 cartridges revealed that 30% and 60% acetonitrile (ACN) eluents contained the highest antibacterial activity against both *E*. *coli* and *S*. *aureus* (S1 Fig). Therefore, both eluents were selected for further purification studies. The peaks 6 (compound A) and 8 (compound B) showed the highest inhibitory activity against *E*. *coli* and *S*. *aureus*, respectively (S2 Fig and Fig 2). We purified both compounds A and B using preparative RP-HPLC from the 30% and 60% SPE fractions. The purification yield of compounds A and B from the culture media was 15 and 10 mg/L, respectively. The purity of the purified compounds was confirmed to be greater than 95% through LC-MS analysis.

### Molecular mass by LC-MS analysis

The purity and mass of the compounds were confirmed using HPLC-MS. The molecular mass of compound A was estimated to be approximately 1519, as [M+H]^+^ and [M+2H]^2+^ were observed at 1519.8 and 760.7 m/z, respectively (S3 Fig). The molecular mass of compound B was estimated to be approximately 1584, as [M+H]^+^ and [M+2H]^2+^ were observed at 1585.1 and 792.9 m/z, respectively (S3 Fig).

### Primary sequence by ESI-MS/MS analysis

**Fig 3:**
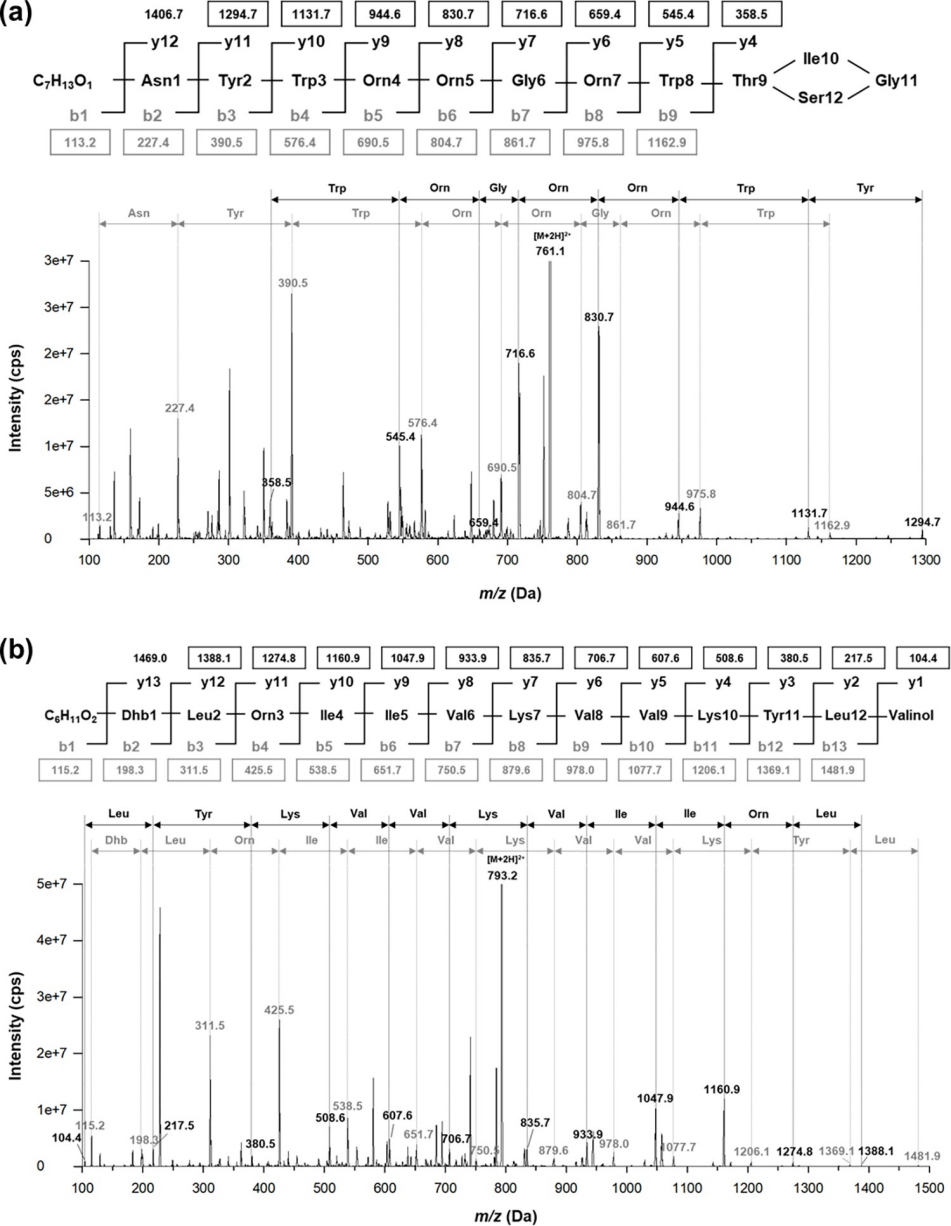
Sequence determination of compound A (a) and compound B (b) using ESI-MS/MS analysis. B-type fragment ions are highlighted in gray, and y-type fragment ions are indicated in black. The mass values that are visible in the spectra are enclosed in boxes.

Tandem ESI-MS/MS analysis was performed to ascertain the primary sequence of each compound. The ion [M+2H]^2+^ of both compounds was chosen as a precursor ion for MS/MS fragmentation. The MS spectra displayed approximate mass values of the fragments of compound A as pairs of b-type ions and y-type ions. Thus, the loss of amino acids is in order from the N-terminus to the C-terminus and vice versa. Indeed, values of b-type ions were detected as 227.4, 390.5, 476.4, 690.5, 804.7, 861.7, 975.8, and 1162.9 m/z pairs with 1294.7, 1131.7, 944.6, 830.7, 716.6, 659.4, 545.4, and 358.5 m/z, which refers to a series loss of amino acids from asparagine to tryptophan (Fig 3A). In compound B, which had a linear structure, all mass values of the fragments except one could be observed in the MS spectra, also in pairs of b-type ions and y-type ions. Likewise, values of b-type ions were found at 198.3, 311.5, 425.5, 538.5, 651.7, 750.5, 879.6, 978.0, 1077.7, 1206.1, 1369.1, and 1481.9 m/z pair with 1388.1, 1274.8, 1160.9, 1047.9, 933.9, 835.7, 706.7, 607.6, 508.6, 380.5, 217.5, and 104.4 m/z, which refers to a series loss of amino acids from dehydrobutyrine (Dhb), dehydration form of threonine to leucine (Fig 3B).

### Chemical structure by NMR analysis

**Fig 4:**
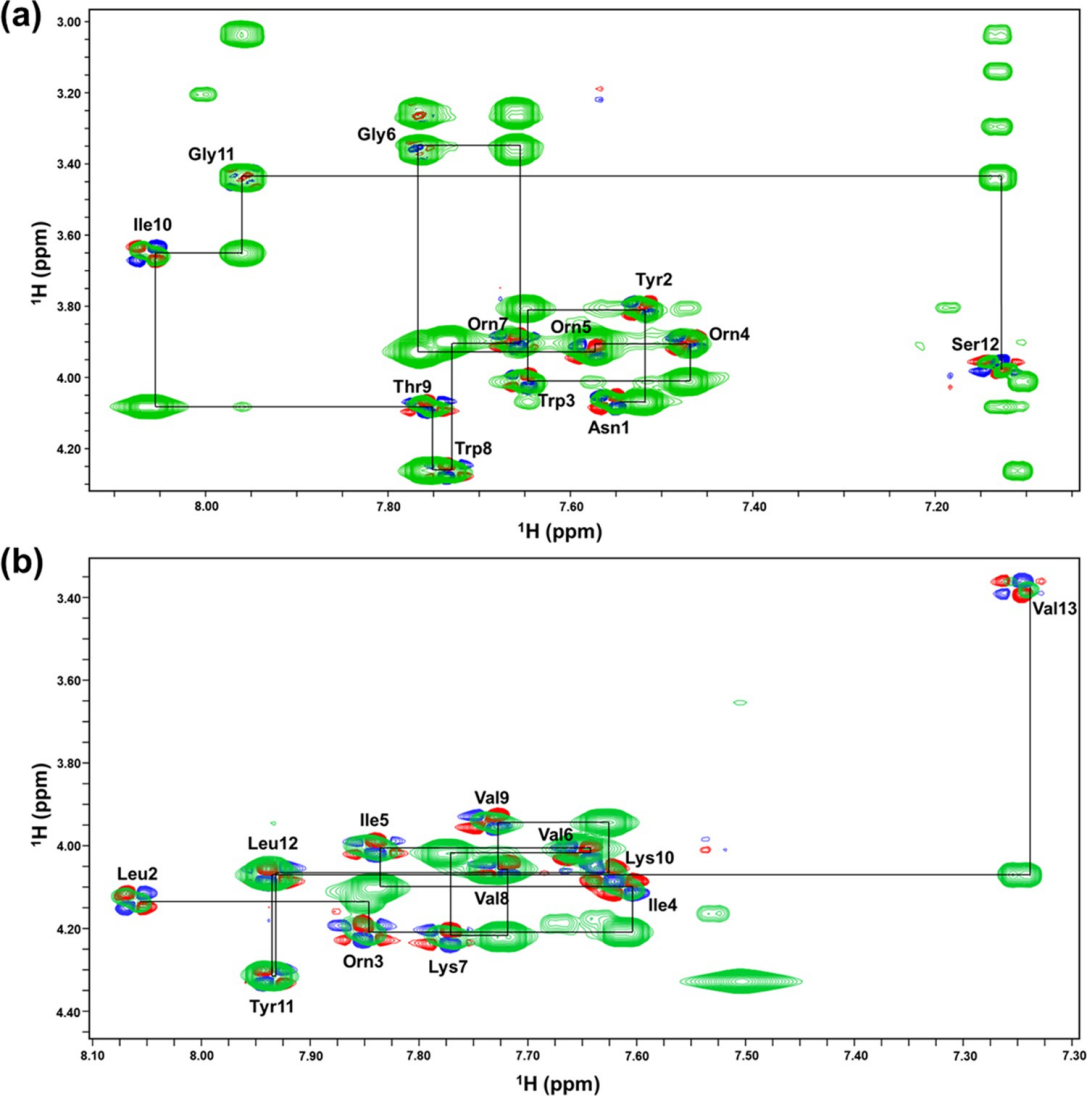
The HN-Hα region of the NOESY and DQF-COSY spectra of compound A (a) and compound B (b). Sequential dαN(*i*, *i + 1*) NOE connectivity for the peptides was determined using ^1^H 2D NOESY. Intra-residue NH-CαH cross peaks are labeled with the corresponding residue name and number.

The exact chemical structure of each compound was determined through the analysis of 2D NMR spectra (S4–S7 Figs). Based on the TOCSY (S4A Fig) and COSY (S4B Fig) spectra, compound A was found to comprise 12 amino acids. Signals of amide proton could be found between 7.12 and 8.05 ppm, and alpha proton were between 3.05 and 4.26 ppm. The NMR analyses identified the presence of one each of Asn, Tyr, Thr, Ile, and Ser; two each of Trp and Gly; and three Orn. The sequential assignment was performed using a 2D NOESY spectrum (Fig 4A). The ^1^H chemical shifts of compound A are summarized in S1 Table. Similarly, the number of amino acids of compound B was confirmed by the TOCSY (S6A Fig) and DQF-COSY (S6B Fig) spectra. It comprises 13 amino acids. Amide proton signals were detected in the range of 7.24 to 8.06 ppm, while alpha proton signals were observed between 3.37 and 4.32 ppm. The NMR analyses revealed the presence of one each of Dhb, Orn, and Tyr; two each of Leu, Ile, and Lys; and four Val, including one valinol. Interestingly, the C-terminus was 2-amino-3-methyl-1-butanol (valinol) that converts the carboxylic group of valines to alcohol, and the first amino acid from the N-terminus was dehydrobutyrine (Dhb), which is a dehydrated form of Thr. The sequential assignment of compound B was performed using a 2D NOESY spectrum (Fig 4B). The ^1^H chemical shifts of compound B are summarized in S2 Table. Based on the NMR analysis, the amino acid sequence of compound A was determined to be FA-Asn-Tyr-Trp-Orn-Orn-Gly-Orn-Trp-Thr-Ile-Gly-Ser (cyclized by ether linkage between Thr and Ser on the C-terminus), which indicated that it was brevicidine (S8A Fig). The amino acid sequence of compound A was determined to be FA-Dhb-Leu-Orn-Ile-Ile-Val-Lys-Val-Val-Lys-Tyr-Leu-valinol, which is identical to brevibacillin (S8B Fig).

### Antibacterial activity

MIC assay was conducted to confirm the antibacterial activity of each compound. A total of four Gram-negative and five Gram-positive bacterial strains, including drug-resistant strains, were subjected to MIC testing. Brevicidine exhibited strong antibacterial activity against Gram-negative bacteria, including a multidrug-resistant *P*. *aeruginosa* strain, with an MIC value of approximately 1 μg/mL. Brevibacillin exhibited significant antibacterial activity against Gram-positive bacterial strains (MIC ranging from 2 to 4 μg/mL) as well as Gram-negative bacteria (MIC ranging from 4 to 64 μg/mL). (Table 1). Melittin, a well-known antimicrobial peptide used as a control, displayed MIC values ranging from 2 to 16 μg/mL, consistent with previously reported data.

**Table 1:** Minimum inhibitory concentration (MIC) of the purified compounds against Gram-negative and Gram-positive bacterial strains.

Strain	MIC (μg/mL)		
	Brevicidine	Brevibacillin	Melittin
**Gram-negative**			
*Escherichia coli* (KCTC 1682)	1	8	8
*Escherichia coli* (ML 35)	1	8	8
*Salmonella typhimurium* (KCTC 1926)	2	16	16
*Pseudomonas aeruginosa* (KCTC 1637)	1	64	16
Multidrug-resistant *Pseudomonas aeruginosa* (CCARM 2095)	1	32	16
Multidrug-resistant *Pseudomonas aeruginosa* (CCARM 2109)	1	32	16
*Acinetobacter baumannii* (KCCM 40203)	8	4	4
*Acinetobacter baumannii* (ATCC BAA 160f)	8	8	4
**Gram-positive**			
*Bacillus subtilis* (KCTC 3068)	16	2	4
*Staphylococcus epidermidis* (KCCM 35494)	128	2	4
*Staphylococcus epidermidis* (KCCM 40416)	128	2	2
*Staphylococcus aureus* (KCTC 1621)	>128	2	4
Methicillin-Resistant *Staphylococcus aureus* (CCARM 3089)	>128	2	2
Methicillin-Resistant *Staphylococcus aureus* (CCARM 3090)	>128	2	4
Methicillin-Resistant *Staphylococcus aureus* (CCARM 3095)	>128	2	4
Vancomycin-resistant *Enterococcus faecium* (ATCC 51559)	>128	2	2
Vancomycin-resistant *Enterococcus faecalis* (ATCC 51575)	>128	4	16

### Time-dependent killing kinetics

**Fig 5:**
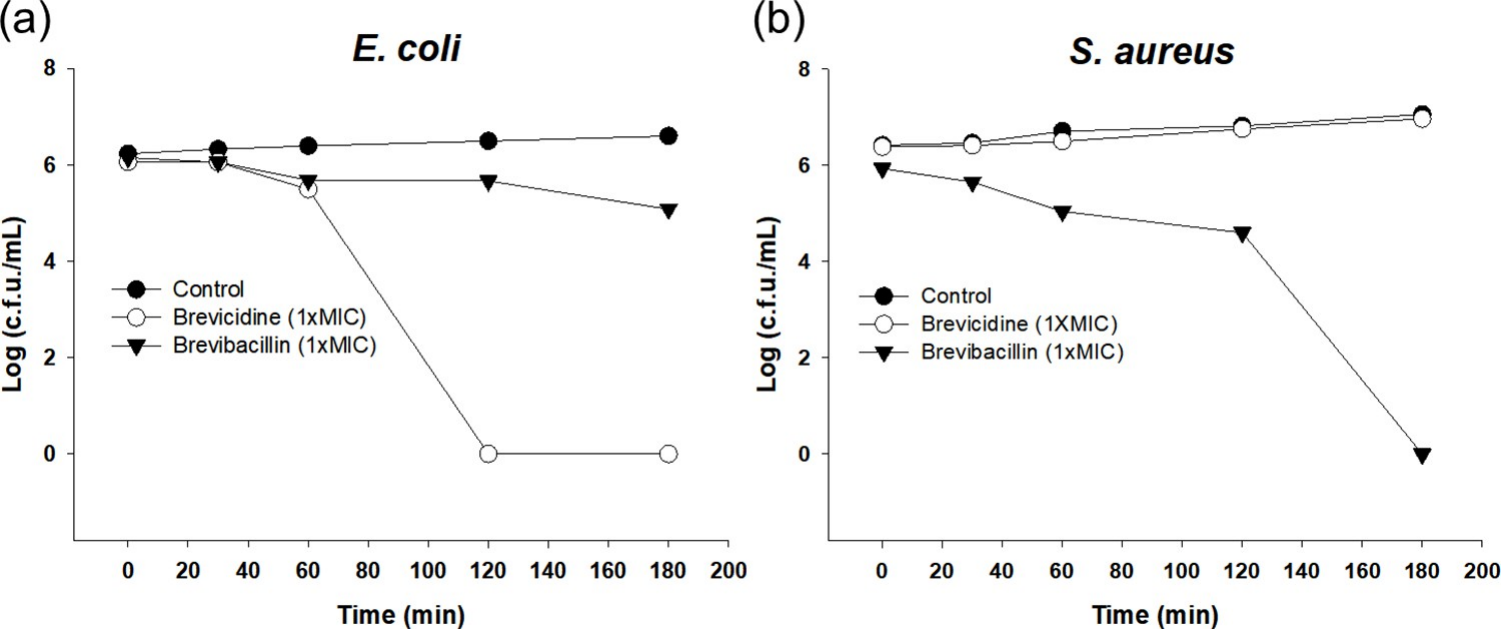
Time-dependent killing kinetics of brevicidine and brevibacillin against *Escherichia coli* (a) and *Staphylococcus aureus* (b) with 1× MIC peptides.

To investigate the relationship between exposure duration to peptides and bacterial survival, we conducted time-dependent killing assays on representative Gram-negative (*E*. *coli*) and Gram-positive (*S*. *aureus*) bacterial species when exposed to brevicidine and brevibacillin. When *E*. *coli* was exposed to 1× MIC of brevicidine, its survival was completely eliminated within 120 min, while this exposure had no impact on the *S*. *aureus* (Fig 5). Conversely, brevibacillin exhibited a slower bactericidal effect on *E*. *coli* but acted more rapidly on *S*. *aureus*. These results indicate that brevicidine and brevibacillin demonstrate cell selectivity against Gram-negative and Gram-positive bacteria, respectively.

### Secondary structure

**Fig 6:**
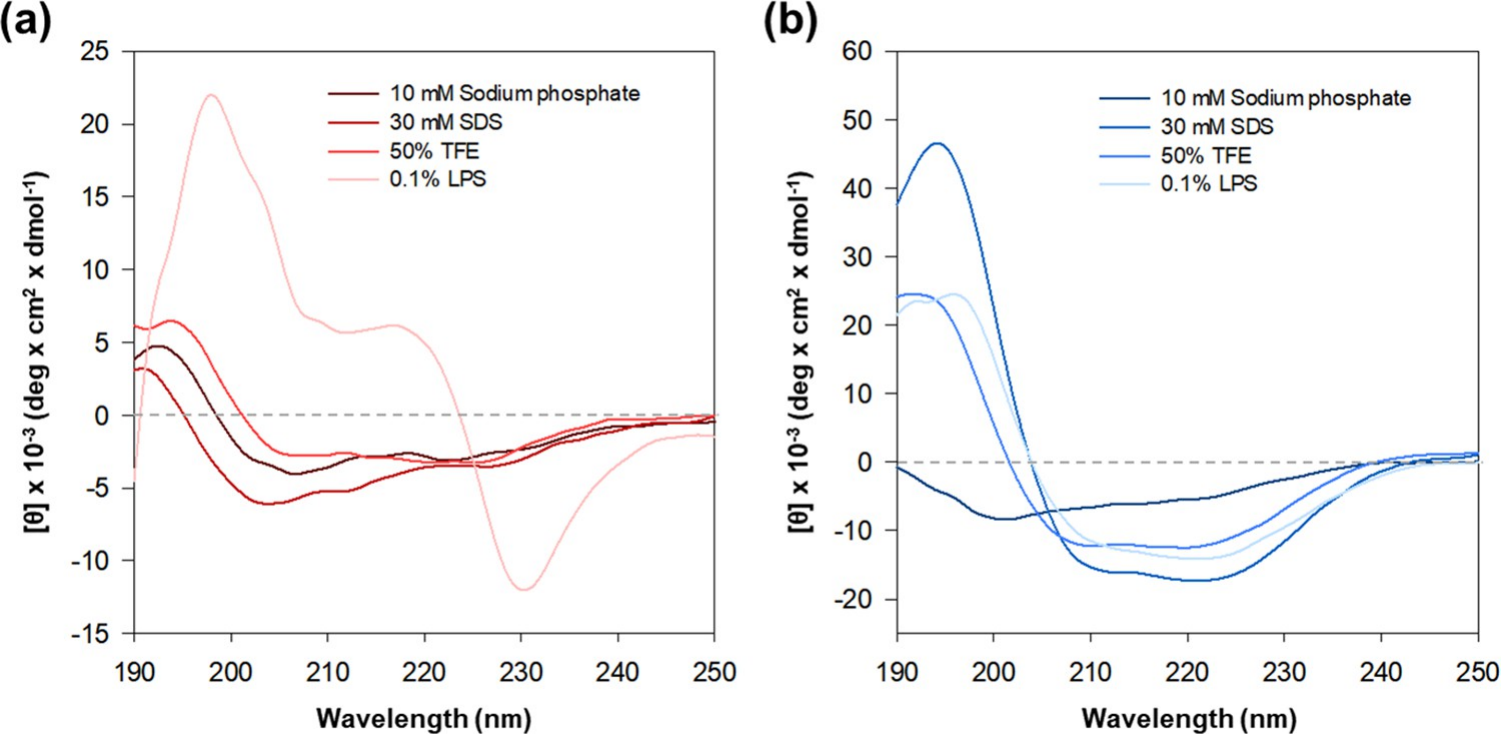
CD spectra of brevicidine (a) and brevibacillin (b) were recorded in different conditions: 10 mM sodium phosphate buffer (pH 6.8), 30 mM sodium dodecyl sulfate (SDS), 50% (v/v) 2,2,2-trifluoroethanol (TFE), and 0.1% (w/v) lipopolysaccharide of *E*. *coli*. The peptide concentration was maintained at 100 μM.

CD spectroscopy was used to determine the secondary structure of each compound. Various conditions were prepared using a buffer based on 10 mM sodium phosphate with a pH of 6.8 (aqueous condition), and adding 30 mM sodium dodecyl sulfate, 50% v/v 2,2,2-trifluoroethanol, and 1% w/v lipopolysaccharide (LPS) of *E*. *coli*. In the LPS solution, Brevicidine exhibited a highly distinctive CD spectrum (Fig 6A). The spectrum in the presence of 0.1% LPS displayed a distinct pattern that did not align with any other known secondary structure motifs, such as random coil, alpha helix, or beta sheet. While, there were observable interactions between brevicidine and LPS, explaining these solely based on CD spectra proved to be challenging. In an aqueous condition, brevibacillin adopts a random coil structure. However, upon the addition of SDS, TFE, and LPS, which serve as membrane mimics and α-helix inducing agents, Brevibacillin undergoes a transition to an α-helix structure (Fig 6B). Subsequently, the structure transformed into an α-helix conformation with a ratio of 27.54% in 50% v/v TFE, 33.48% in 0.1% w/v LPS, and 43.14% in 30 mM SDS, (S3 Table).

### Stability of brevicidine and brevibacillin

**Fig 7:**
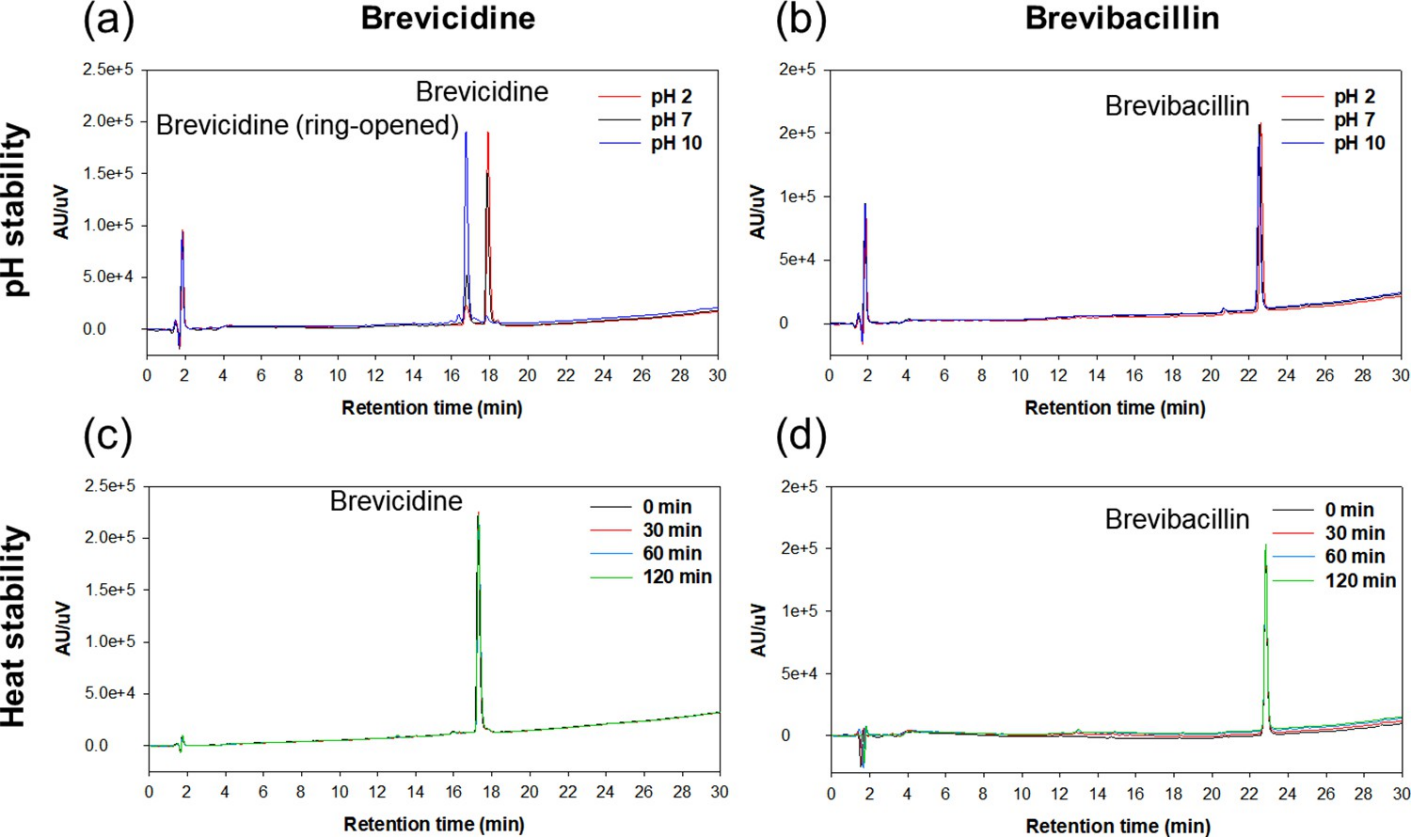
The stability of brevicidine and brevibacillin under different pH and high temperature condition. Brevicidine (a) and brevibacillin (b) at different pH 2, 7, and 10. Brevicidine (c) and brevibacillin (d) with 80°C incubation for 120 min.

The stability of the peptides in different pH buffers and at high temperature was analyzed using RP-HPLC (Fig 7). There were no changes in HPLC profiles with the various pH values and the 80°C incubation for both brevicidine and brevibacillin, except for pH 10 in the case of brevicidine (Fig 7A). The HPLC peak of brevicidine shifted to an earlier time point (18 min to 16.5 min) at pH 10. The earlier peak exhibited a molecular weight of brevicidine+18 (S9 Fig), indicating that the C-terminal ester-linked macrocycle of brevicidine had undergone hydrolysis. Generally, ester bonds are easily hydrolyzed under basic conditions. Brevibacillin, on the other hand, lacks such a macrocycle in its structure, and therefore, no changes occurred at basic pH.

### Hemolytic activity

**Fig 8:**
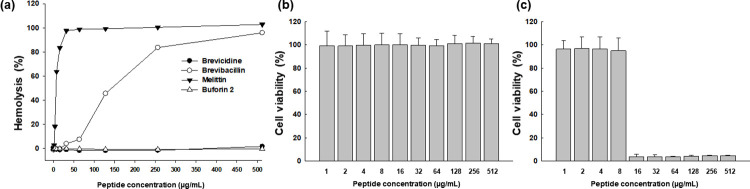
Cytotoxicity of brevicidine and brevibacillin. (a) Hemolytic activity assessed using red blood cells from sheep. Cell viability of brevicidine (b) and brevibacillin (c) measured using the MTT assay for RAW264.7 cells.

Hemolysis is characterized by the liberation of hemoglobin following damage to blood cells. Melittin, an extensively recognized antimicrobial peptide (AMP) renowned for its substantial cytotoxicity, was employed as a positive control. In contrast, buforin II, an AMP derived from the stomach tissue of the Asiatic toad (*Bufo gargarizans*) and lacking any hemolytic activity, was used as the negative control. As depicted in Fig 8A, melittin induced a hemolysis level of 97.7% at 32 μg/mL, whereas buforin II displayed no hemolytic activity. Brevicidine exhibited no hemolysis even up to 512 μg/mL, similar to the results of the negative control. This suggests that it lacked cytotoxicity towards sheep red blood cells. On the contrary, brevibacillin exhibited an increasing level of hemolysis with higher concentrations, ranging from 45.5% at 128 μg/mL to 95.9% at 512 μg/mL (Fig 8A).

### Cytotoxicity

The cytotoxicity of the purified compounds was assessed using the MTT assay (Fig 8B and 8C). The cell viability of RAW264.7 cells remained at 100% even up to a concentration of 512 μg/mL for brevicidine, suggesting its non-toxic nature towards mammalian cell lines. This finding closely paralleled the outcomes of the hemolysis assay. When treated with brevibacillin, cell viability exhibited a sharp decline, plummeting from 95% to 4% within the concentration range of 8 to 16 μg/mL. This points to a pronounced cytotoxic effect on mammalian cell lines (Fig 8C).

### Cytoplasmic membrane electrical potential

**Fig 9:**
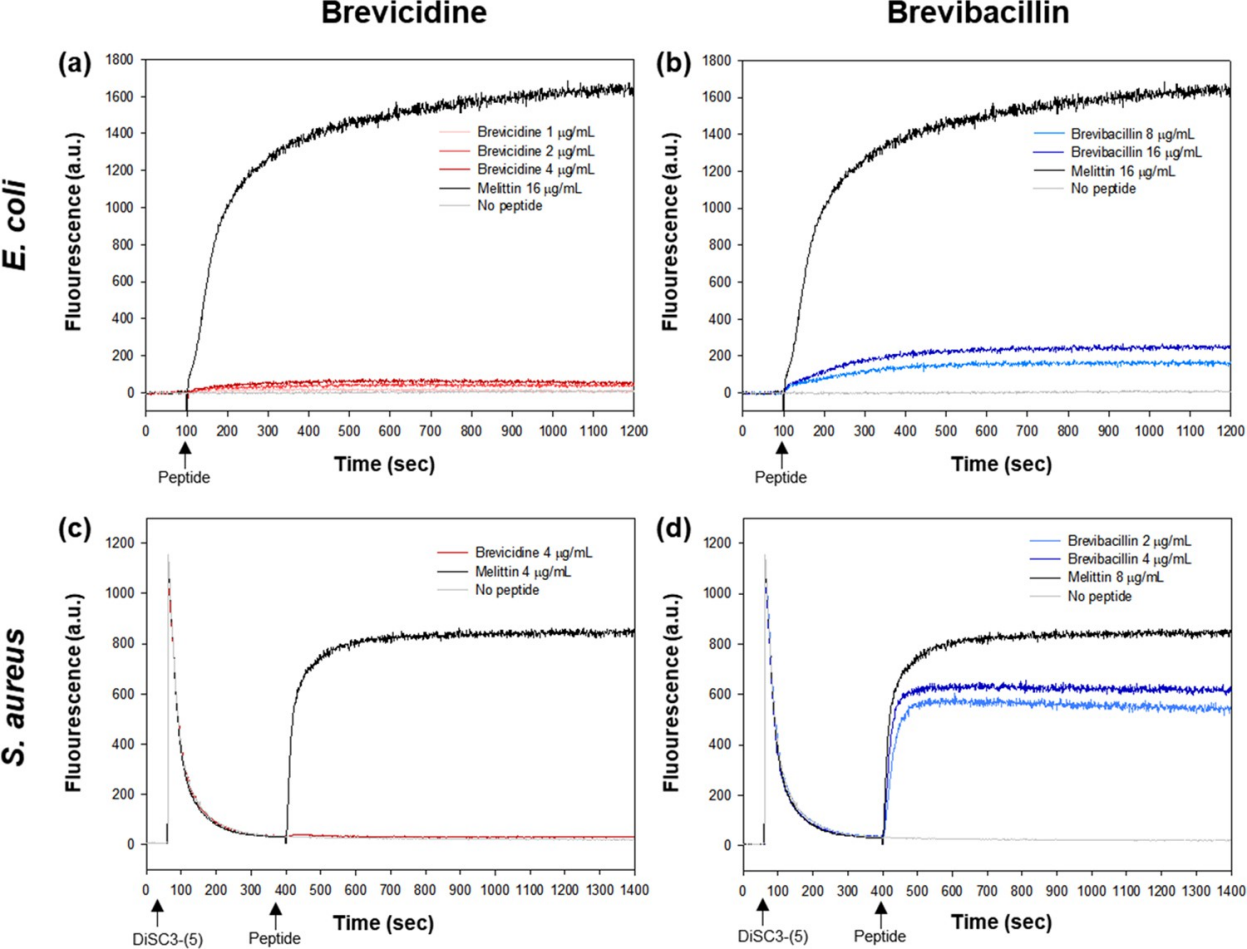
Membrane depolarization of *E*. *coli* and *S*. *aureus* induced by brevicidine and brevibacillin. (a) *E*. *coli* treated with brevicidine, (b) *E*. *coli* treated with brevibacillin, (c) *S*. *aureus* treated with brevicidine and (d) *S*. *aureus* treated with brevibacillin.

To verify the disruption of membranes caused by the compounds, a membrane depolarization test was conducted. This assay utilizes DiSC3-(5) dye, which enhances fluorescence intensity upon alteration of the membrane potential due to cytoplasmic membrane damage. In the case of *E*. *coli*, a rapid increase in fluorescence intensity was observed after treating melittin—a peptide known for its capability to disrupt bacterial cell membranes—at a concentration of 2× MIC (16 μg/mL). This phenomenon indicates membrane damage and subsequent dye leakage. Despite being treated with brevicidine up to 4× MIC (4 μg/mL), there was no increase in fluorescence intensity. This indicates that the treatment with brevicidine did not alter the membrane potential (Fig 9A). On the contrary, a gradual increase in fluorescence intensity was observed when brevibacillin was added (Fig 9B). Brevibacillin interacted with the cell membrane of *E*. *coli*; however, its effect was significantly less pronounced compared to that of melittin. With *S*. *aureus*, a sudden increase in fluorescence intensity was observed upon melittin treatment at 2× MIC (16 μg/mL). The fluorescence intensity for brevicidine was identical to that of the negative control, indicating the absence of antibacterial activity against *S*. *aureus* (Fig 9C). Brevibacillin exhibited a sharp increase in fluorescence intensity, which differed from the result observed in *E*. *coli*. The cell membrane of *S*. *aureus* caused rapid dye leakage from the cell, indicating a faster and more extensive impact compared to that in *E*. *coli* (Fig 9D).

### SEM analysis

**Fig 10:**
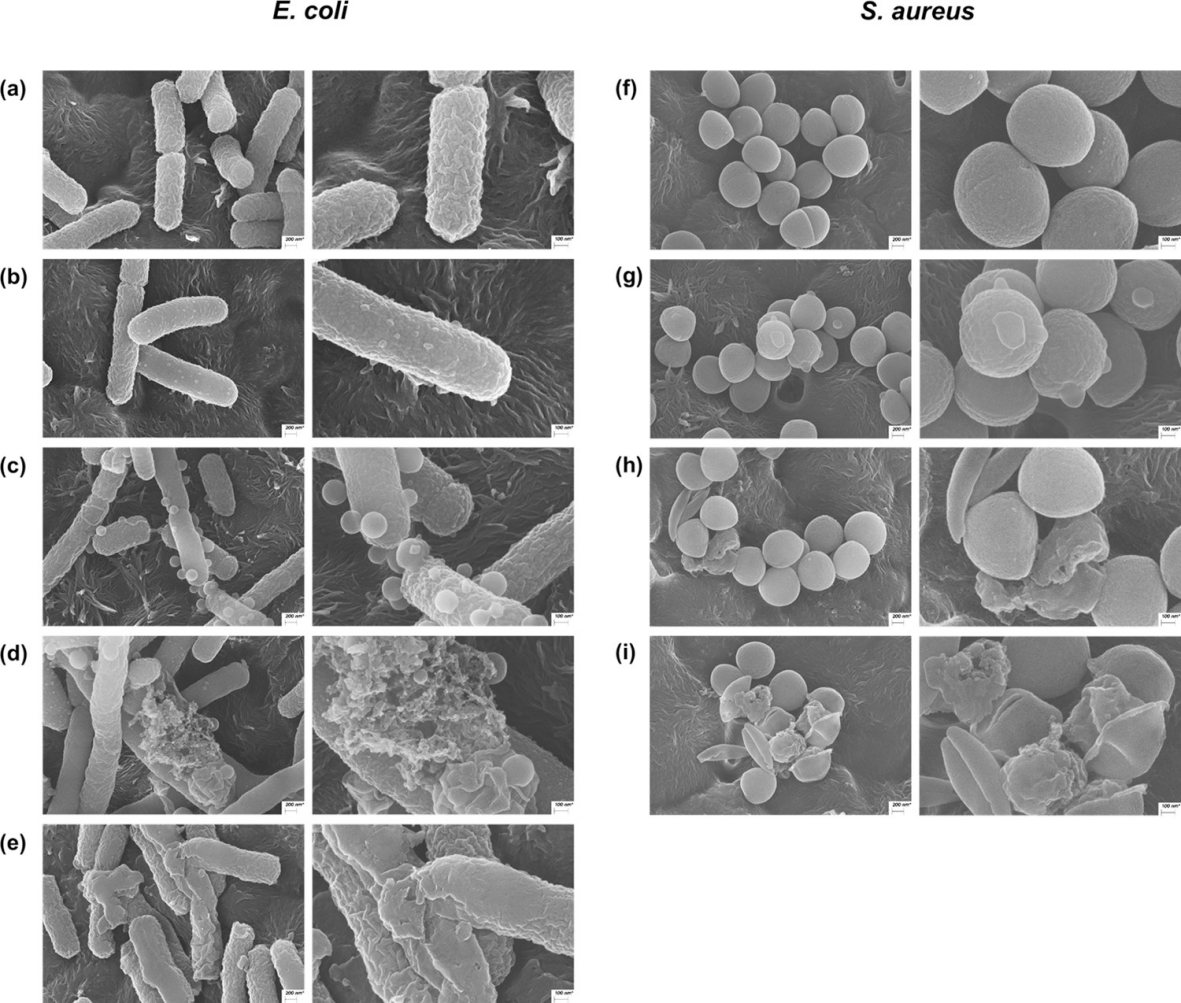
SEM images of *E*. *coli* treated with the peptides. Control (a), brevicidine at 0.5× MIC (b), 1× MIC (c), and 2× MIC (d), as well as brevibacillin at 0.5× MIC (e). SEM images of *S*. *aureus* treated with brevibacillin. Control (f), brevibacillin at 0.5× MIC (g), 1× MIC (h), and 2× MIC (i). The left column presents images at 25,000× magnification. The right column features enlarged sections from the left images.

The untreated *E*. *coli* displayed a rod-shaped structure with a wrinkled surface, as shown in Fig 10A. Upon addition of 0.5× MIC of brevicidine, the surface wrinkles seemed to diminish, giving rise to small swellings (Fig 10B). At 1× MIC, they enlarged and developed blister-like protrusions (Fig 10C). Finally, at 2× MIC, cell rupture was observed (Fig 10D). These results indicate that brevicidine affects the cell membrane of *E*. *coli*, leading to bacterial death. In the brevibacillin treatments, the observed surface of *E*. *coli* became smooth, and the rod shape was disrupted at a concentration of 0.5× MIC (Fig 10E). *S*. *aureus* exhibited round-shaped cells, as evident in the untreated bacteria (Fig 10F). Certain swellings were observed at 0.5× MIC of brevibacillin treatment (Fig 10G), although the shape differed from that of *E*. *coli*. At concentrations of 1× MIC or higher of brevibacillin, *S*. *aureus* lost its round shape and underwent severe deformation, destruction, and shrinking (Fig 10H and 10I).

## Discussion

### Structural analysis

Brevicidine (MW: 1519 Da) was initially discovered from *B*. *laterosporus* DSM25. It was identified as a cationic non-ribosomal peptide, a lipodepsipeptide 4-Methyl-Hexanoyl-D-Asn-D-Tyr-D-Trp-D-Orn-Orn-Gly-D-Orn-Trp-Thr-Ile-Gly-Ser [40]. A family of brevicidine, named brevicidine B (MW: 1503 Da), which contains a single substitution (Tyr2 to Phe2) in the amino acid sequence of the linear part of brevicidine, was detected from the same strain, namely *B*. *laterosporus* DSM25 [30]. According to the previous study [40], brevicidine has a partial cycle structure in its C-terminus of amino acid sequence. Brevicidine is widely recognized as a cyclic compound and poses challenges in acquiring fragmentation spectra due to its structural stability. With regard to brevicidine, preliminary experimentation is necessary before conducting MS analysis to ensure the opening of the ring structure. The pairs of b-type ion and y-type ion that refer to a series loss of amino acids from asparagine to tryptophan were successfully detected.

Brevibacillin (MW: 1583 Da) containing 13 amino acids and a C6 fatty acid at the N-terminus was identified from *B*. *laterosporus* OSY-I1 [28]. Through structural analysis using MS/MS and NMR, it was identified as a cationic lipopeptide, FA-Dhb-Leu-Orn-Ile-Ile-Val-Lys-Val-Val-Lys-Tyr-Leu-valinol, where FA is 2-hydroxy-3-methylpentanoic acid, Dhb is dehydrobutyrine, and valinol is 2-amino-3-methyl-1-butanol. A family of brevibacillin, named brevibacillin V (MW: 1570 Da), which contains a single substitution (Ile5 to Val5) in the amino acid sequence, was identified from *B*. *laterosporus* fmb70 [41]. Brevibacillin I (MW: 1597, with a single substitution of Val8 to Ile8) and brevibacillin 2V (MW: 1555, with a dyadic substitution of Ile4 to Val4 and Ile5 to Val5), were isolated from *B*. *laterosporus* DSM 25 [42]. In the MS/MS analysis of brevibacillin, the detection of pairs of b-type and y-type ions exhibiting a sequential loss of amino acids from dehydrobutyrine (Dhb), a dehydrated form of threonine, to leucine was observed.

### Antimicrobial activity

Brevicidine exhibited significant antibacterial activity against Gram-negative bacteria, including multidrug-resistant *P*. *aeruginosa*, with MIC values of approximately 1 μg/mL. A noteworthy aspect is that brevicidine exhibited no antibacterial activity against Gram-positive bacterial strains. This observation suggests a specific selectivity that could be utilized for the development of antibiotics specifically targeting Gram-negative bacteria. The findings are in line with the previous studies, highlighting the robust antibacterial activity of brevicidine against Gram-negative bacteria and antimicrobial-resistant *Enterobacteriaceae* pathogens [40, 43]. Furthermore, brevicidine has been documented to possess strong antibiofilm activity against *Enterobacteriaceae* pathogens [43]. Brevicidine B exhibited a broad spectrum of antimicrobial and bactericidal activity against both Gram-negative and Gram-positive bacteria [30]. Brevibacillin demonstrated remarkable antibacterial activity against Gram-positive bacteria, with MIC values of approximately 2 μg/mL. While its selectivity might not be as high as that of brevicidine, this outcome remains noteworthy. Brevibacillin exhibited low MICs against Gram-positive bacteria such as methicillin-resistant *S*. *aureus* and vancomycin-resistant enterococci, suggesting its potential as an antibiotic. These results are consistent with other studies that emphasize the significant antimicrobial activity of brevibacillin against methicillin-resistant *S*. *aureus*, *Listeria monocytogenes*, *Bacillus cereus*, phytopathogenic fungi, and foodborne pathogenic bacteria [28, 41]. These results present both brevicidine and brevibacillin as viable and effective alternatives for combating microbial infections.

### Antibacterial mode of action

A membrane depolarization assay was conducted to elucidate the mode of action of each compound on bacterial membranes. The findings for brevicidine suggest that it did not alter the membrane potential of *E*. *coli*. The impact of brevicidine on the bacterial surface was visualized using SEM. Brevicidine induced the formation of balloon-shaped blisters on the cell wall, in a concentration-dependent manner, and at sufficiently high concentrations led to cell rupture. A similar phenomenon was documented in a previous report where *E*. *coli* exposed to arginine-rich histones (H3 and H4) exhibited similar behavior. These histones adhered to the cell surface, leading to subsequent disruption of the cell membrane structure. The result was the identification of irregular bacterial surfaces and cell aggregation in the cells treated with histones H3 and H4 [44].

CD spectroscopy unveiled an atypical spectrum of brevicidine with LPS, implying a level of interaction between brevicidine and LPS. This finding suggests that brevicidine possesses a strong binding capability to LPS. This attribute could contribute to the specific antibacterial activity of brevicidine against Gram-negative bacteria. These results align with a prior study that demonstrated the binding ability of brevicidine to LPS and its interaction with phosphatidylglycerol and cardiolipin within the inner membrane. This interaction led to the disruption of the proton motive force [43, 45]. The bactericidal activity of brevicidine is attributed to several mechanisms involved in metabolic perturbation, including the dissipation of the proton motive force, inhibition of ATP biosynthesis, dehydrogenation of NADH, accumulation of reactive oxygen species (ROS), and eventual cell death [43]. Furthermore, brevicidine has the capacity to inhibit protein synthesis by targeting tRNA ligase and ribosomal protein synthesis [43]. Additionally, brevicidine B increased membrane disruptive capacity on Gram-positive pathogens and disrupted the proton motive force of cells against Gram-negative bacteria [30]. The fluorescence microscopy assay revealed that brevicidine does not induce bacterial death by disrupting bacterial membranes. Intriguingly, following a 15-minute treatment with brevicidine (1× MIC), surface wrinkles were observed on the bacteria. Furthermore, SEM images illustrated that a significant portion of the bacteria had fractured after a 120 min treatment with brevicidine at 1× MIC. The results from a previous report, specifically the time-killing assay, which indicated that brevicidine initiated bactericidal activity after 30 min [43]. Based on membrane targeting assays, it can be inferred that brevibacillin has the capability to disturb the cell membrane, resulting in the demise of the pathogen. Likewise, SEM images revealed that the bacterial surface underwent substantial damage and shriveling when exposed to brevibacillin. The CD spectrum indicated that brevibacillin adopted an amphipathic α-helical structure at the cell membrane, facilitating its penetration through the bacterial cell wall. Moreover, it has been demonstrated that the disruption of the cytoplasmic membrane in *S*. *aureus* is attributed to the binding of brevibacillin with lipoteichoic acid [46]. Moreover, brevibacillin disrupts cytoplasmic membrane of *S*. *aureus* by increasing its permeability, depolarization and potassium leakage [46].

Brevicidine exhibited no hemolytic activity or cytotoxicity up to a concentration of 512 μg/mL when tested with sheep red blood cells and RAW264.7 cells. These outcomes are consistent with another study that reported the absence of hemolytic activity and cytotoxicity even at high concentrations of brevicidine B [30]. Based on these findings, it appears that brevicidine holds promise as an antibiotic candidate. Its substantial antibacterial efficacy against Gram-negative bacteria, including multidrug-resistant strains, along with its favorable selectivity and lack of cytotoxicity against mammalian cells, suggests its potential suitability for antibiotic use. Conversely, brevibacillin exhibited slightly elevated levels of hemolytic and cytotoxic activities in the *in vitro* assays, potentially attributable to its mode of action. Nevertheless, brevibacillin still demonstrates substantial antimicrobial potency against multidrug-resistant bacteria. Consequently, additional research is warranted to mitigate its cytotoxic effects and enhance its safety profile. For instance, through the substitution of two isoleucines of brevibacillin 2V with valines, the hemolysis was eliminated up to a concentration of 128 μg/mL [42]. It has also been established that the hemolytic activity of lipopeptides is linked to the length of their fatty acid chain [47–49]. This implies that by making modifications to the amino acid sequence of brevibacillin, its cytotoxicity could potentially be reduced, thereby rendering it more suitable for future antibiotic applications. The good plasma stability of brevicidine and brevibacillin offers them as promising antimicrobial candidates for treating bacterial infections in both humans and animals [43].

## Conclusion

In this study, both brevicidine and brevibacillin were isolated and purified from a novel strain of *B*. *laterosporus* TSA31-5, obtained from red clay soil. *B*. *laterosporus* strain simultaneously produced brevicidine and brevibacillin, both of which selectively acted on gram-negative and positive bacteria. Their molecular weights, amino acid sequences, and complete chemical structures were successfully determined. Brevicidine exhibited specific antibacterial activity targeting Gram-negative bacteria, even against drug-resistant strains. Notably, it displayed an affinity for binding with LPS and influenced bacterial surfaces, inducing the formation of balloon-shaped blisters that ultimately led to cell lysis. Moreover, brevicidine demonstrated no hemolytic or cytotoxic effects at concentrations up to 512 μg/mL. These findings highlight the potential utility of brevicidine as a safe and effective antimicrobial agent, particularly against Gram-negative pathogens. Brevibacillin exhibited notable antibacterial efficacy against both Gram-negative and Gram-positive bacteria, encompassing drug-resistant strains. It adopted an amphipathic α-helical structure in a membrane mimic environment. The disturbance of the cell membrane led to the collapse of the cells. This mode of action resulted in slightly elevated hemolytic and cytotoxic activities, indicating potential limitations for its clinical application as an effective medication. However, adjustments to the amino acid sequence of brevibacillin could potentially mitigate its hemolytic and cytotoxic effects while maintaining high antimicrobial efficiency. These peptides hold promise for further development as potentially valuable and effective antimicrobial agents to address the challenge of antibiotic-resistant infections. Our findings pave the way for further studies on the clinical applications of the antimicrobial peptides to combat prevalent bacterial infections. Modifications to the amino acid sequence of brevibacillin could potentially reduce its cytotoxicity, thereby rendering it more suitable for future antibiotic applications. Additionally, the synthesized analogues of brevicidine and brevibacillin could demonstrate more hydrolytically stable and potent antibacterial activity both in *in vitro* and *in vivo* infection models.

## Supporting information

S1 Table The ^1^H and ^13^C chemical shifts of compound A (brevicidine). (PDF)

S2 Table The ^1^H and ^13^C chemical shifts of compound B (brevibacillin). (PDF)

S3 Table The [θ]_222_ and percent of α-helical contents of brevibacillin from CD analysis in various buffer conditions. (PDF)

S1 Fig The antibacterial activity of solid phase extraction (SPE) eluents with different concentrations of acetonitrile (0, 30, 60, and 100%) against *Escherichia coli* (a) and *Staphylococcus aureus* (b) was assessed.
(PDF)

S2 Fig The RP-HPLC chromatogram (a) and antibacterial activity (b) of the compounds in the 30% eluent of SPE purification are shown. Peaks 7 and 8 in the chromatogram correspond to compounds A and B, respectively.
(PDF)

S3 Fig The LC-MS analysis of compound A and B is depicted. RP-HPLC chromatograms (a and c) along with MS spectra (b and d) of the purified compounds A and B, respectively, are presented.
(PDF)

S4 Fig ^1^H 2D TOCSY (a) and DQF-COSY (b) NMR spectra of compound A.
(PDF)

S5 Fig ^1^H-^13^C HSQC (a), HMBC (b), and fatty acid chain region of HMBC (c) NMR spectrum of compound A.
(PDF)

S6 Fig ^1^H 2D TOCSY (a) and DQF-COSY (b) NMR spectra of compound B.
(PDF)

S7 Fig ^1^H-^13^C HSQC (a), HMBC (b), and fatty acid chain region of HMBC (c) NMR spectrum of compound B.
(PDF)

S8 Fig Chemical structure of brevicidine (a) and brevibacillin (b).
(PDF)

S9 Fig LC-MS spectra of brevicidine (a) and ring-opened brevicidine (b) hydrolyzed at pH10.
(PDF)
