# Supplementary material for: New strain Brevibacillus laterosporus TSA31-5 produces both brevicidine and brevibacillin, exhibiting distinct antibacterial modes of action against Gram-negative and Gram-positive bacteria
Source: PLoS One. 2024 Apr 1;19(4):e0294474. doi: 10.1371/journal.pone.0294474 (PMC10984550; doi:10.1371/journal.pone.0294474)
Supplement: S1 Table — (PDF) [file pone.0294474.s001.pdf]

S1 Table. The ^{1}H and ^{13}C chemical shifts of compound A (brevicidine)

Sequence	Position	δ_H (ppm)	δ_C (ppm)	Sequence	Position	δ_H (ppm)	δ_C (ppm)
Asn1	HN	7.55		Orn7	HN	7.65	
	1	4.07	52.03		37	3.90	54.08
	2	1.89, 2.05	39.39		38	0.93	31.87
	3		52.10		39	0.84	31.87
	4		174.73		40	2.12	41.10
	HD	6.52, 6.99			41		173.50
Tyr2	HN	7.52			HZ	7.14	
	5	3.81	57.50	Trp8	HN	7.73	
	6	2.14, 2.32	38.70		42	4.26	55.45
	7		132.49		43	2.50, 2.62	30.50
	8	6.38	132.67		44		130.02
	9	6.09	117.31		45		130.03
	10		158.32		46	7.10	121.07
	11	6.09	117.31		47	6.49	120.73
	12	6.38	132.67		48	6.59	123.46
	13		173.91		49	6.86	113.89
Trp3	HN	7.65			50		130.02
	14	4.01	56.48		51	6.63	126.54
	15	2.55, 2.70	29.82		52		173.91
	16		112.80		HE	10.32	
	17		130.02	Thr9	HN	7.75	
	18	7.10	120.73		53	4.08	56.48
	19	6.53	121.07		54	4.42	72.54
	20	6.60	123.47		55	0.62	16.49
	21	6.87	113.89		56		169.40
	22		130.02	lle10	HN	8.05	
	23	6.70	126.20		57	3.65	61.26
	24		174.32		58	1.38	36.99
	HE	10.32			59	0.61	31.53
Orn4	HN	7.47			60	0.38	18.20
	25	3.90	54.36		61	1.04	27.08
	26	1.27	31.53		62		173.91
	27	1.09	31.53	Gly11	HN	7.96	
	28	2.31	38.70		63	3.05, 3.44	45.88
	29		173.50		64		171.86
	HZ	7.21		Ser12	HN	7.12	
Orn5	HN	7.57			65	3.97	57.16
	30	3.93	54.08		66	3.14, 3.29	63.99
	31	1.23	32.21		67		169.40
	32	1.06	32.21		OH	4.69	
	33	2.29	41.10	Fatty acid	1'		175.55
	34		173.91		2'	1.60	35.63
	HZ	7.21			3'	0.80, 1.04	34.26
Gly6	HN	7.76			4'	0.64	31.53
	35	3.27, 3.35	44.17		5'	0.35	13.75
	36		173.91		6'	0.80	35.97
					7'	0.35	21.61
