# Supplementary material for: New strain Brevibacillus laterosporus TSA31-5 produces both brevicidine and brevibacillin, exhibiting distinct antibacterial modes of action against Gram-negative and Gram-positive bacteria
Source: PLoS One. 2024 Apr 1;19(4):e0294474. doi: 10.1371/journal.pone.0294474 (PMC10984550; doi:10.1371/journal.pone.0294474)
Supplement: S2 Table — (PDF) [file pone.0294474.s002.pdf]

S2 Table. The ^{1}H and ^{13}C chemical shifts of compound B (brevibacillin)

Sequence	Position	δ_H (ppm)	δ_C (ppm)	Sequence	Position	δ_H (ppm)	δ_C (ppm)
Dhb1	HN	9.12		Val8	HN	7.72	
	1		134.43		39	4.06	60.19
	2	5.66	120.69		40	1.76	33.20
	3	1.58	15.76		41	0.61	21.23
	4		167.24		42	0.61	21.23
Leu2	HN	8.06			43		173.80
	5	4.13	54.38	Val9	HN	7.73	
	6	1.39	43.11		44	3.94	60.54
	7	1.50	27.04		45	1.76	33.20
	8	0.71	26.36		46	0.62	20.89
	9	0.68	23.97		47	0.62	20.89
	10		175.03		48		173.39
Orn3	HN	7.85		Lys10	HN	7.63	
	11	4.21	54.73		49	4.07	55.07
	12	1.48, 1.52	31.83		50	1.16, 1.26	34.56
	13	1.34, 1.39	26.36		51	0.88	24.65
	14	2.60	41.40		52	1.25	29.43
	15		173.80		53	2.50	41.74
	HZ	7.50			54		173.80
Ile4	HN	7.60			HZ	7.50	
	16	4.10	59.17	Tyr11	HN	7.93	
	17	1.54	39.69		55	4.32	57.12
	18	0.86	27.04		56	2.44, 2.67	40.37
	19	0.61	13.71		57		133.20
	20	1.19	27.04		58	6.83	132.99
	21		173.80		59	6.46	117.61
Ile5	HN	7.83			60		158.63
	22	4.01	59.85		61	6.46	117.61
	23	1.54	39.00		62	6.83	132.99
	24	0.88	27.38		63		173.80
	25	0.60	14.05	Leu12	HN	7.93	
	26	1.26	27.38		64	4.07	54.04
	27		173.80		65	1.23	43.79
Val6	HN	7.64			66	1.17	26.70
	28	4.02	60.88		67	0.65	26.02
	29	1.76	33.20		68	0.60	24.65
	30	0.64	20.89		69		174.62
	31	0.64	20.89	Val13	HN	7.24	
	32		173.39		70	3.37	58.49
Lys7	HN	7.77			71	1.64	31.14
	33	4.22	54.73		72	0.65	22.60
	34	1.32, 1.46	34.90		73	0.65	22.60
	35	1.10	24.99		74	1.64	31.14
	36	1.32	29.43		OH	4.33	
	37	2.53	41.74	Fatty acid	1'		175.44
	38		174.21		2'	3.65	77.97
	HZ	7.50			3'	1.53	41.05
					4'	0.71	18.16
					5'	0.96	26.02
					6'	0.64	14.74
					OH	5.44	
