# Supplementary material for: New strain Brevibacillus laterosporus TSA31-5 produces both brevicidine and brevibacillin, exhibiting distinct antibacterial modes of action against Gram-negative and Gram-positive bacteria
Source: PLoS One. 2024 Apr 1;19(4):e0294474. doi: 10.1371/journal.pone.0294474 (PMC10984550; doi:10.1371/journal.pone.0294474)
Supplement: S3 Table — (PDF) [file pone.0294474.s003.pdf]

S3 Table. The $[\theta]_{222}$ and percent of α-helical contents of brevibacillin from CD analysis in various buffer conditions

Buffer	$[\theta]_{222}$	% α-helix
10 mM sodium phosphate	-5,229.08	random coil
30 mM SDS	-17,236.83	43.14
50% TFE	-12,088.83	27.54
0.1% LPS	-14,047.33	33.48
