# Supplementary material for: New strain Brevibacillus laterosporus TSA31-5 produces both brevicidine and brevibacillin, exhibiting distinct antibacterial modes of action against Gram-negative and Gram-positive bacteria
Source: PLoS One. 2024 Apr 1;19(4):e0294474. doi: 10.1371/journal.pone.0294474 (PMC10984550; doi:10.1371/journal.pone.0294474)
Supplement: S1 Fig — The antibacterial activity of solid phase extraction (SPE) eluents with different concentrations of acetonitrile (0, 30, 60, and 100%) against Escherichia coli (a) and Staphylococcus aureus (b) was assessed. (PDF) [file pone.0294474.s004.pdf]

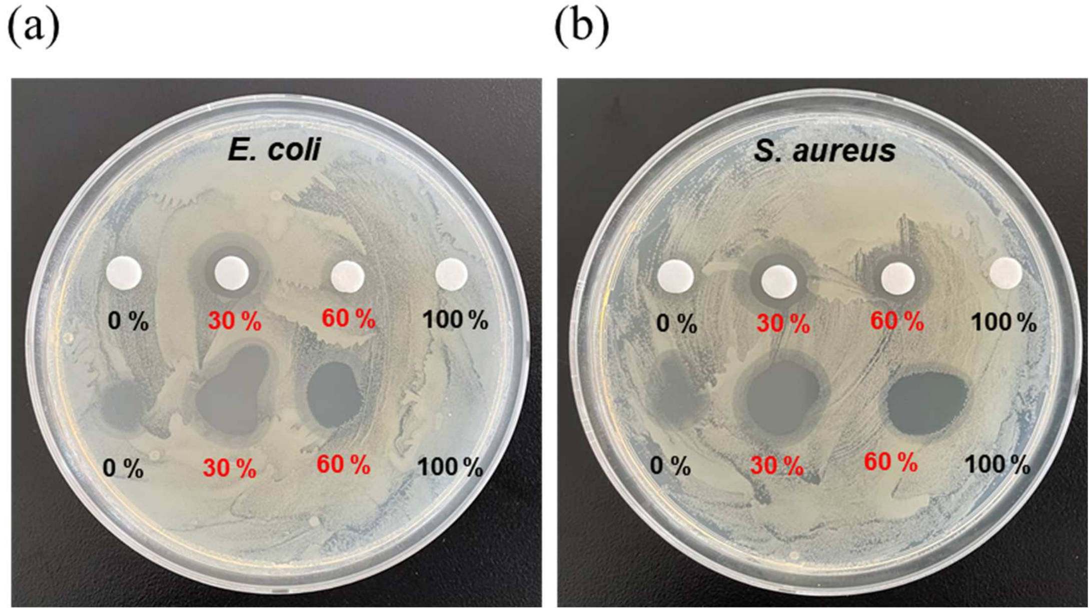

S1 Fig. The antibacterial activity of solid phase extraction (SPE) eluents with different concentrations of acetonitrile (0, 30, 60, and 100%) against *Escherichia coli* (a) and *Staphylococcus aureus* (b) was assessed.
