# Supplementary material for: New strain Brevibacillus laterosporus TSA31-5 produces both brevicidine and brevibacillin, exhibiting distinct antibacterial modes of action against Gram-negative and Gram-positive bacteria
Source: PLoS One. 2024 Apr 1;19(4):e0294474. doi: 10.1371/journal.pone.0294474 (PMC10984550; doi:10.1371/journal.pone.0294474)
Supplement: S2 Fig — The RP-HPLC chromatogram (a) and antibacterial activity (b) of the compounds in the 30% eluent of SPE purification are shown. Peaks 7 and 8 in the chromatogram correspond to compounds A and B, respectively. (PDF) [file pone.0294474.s005.pdf]

(a)

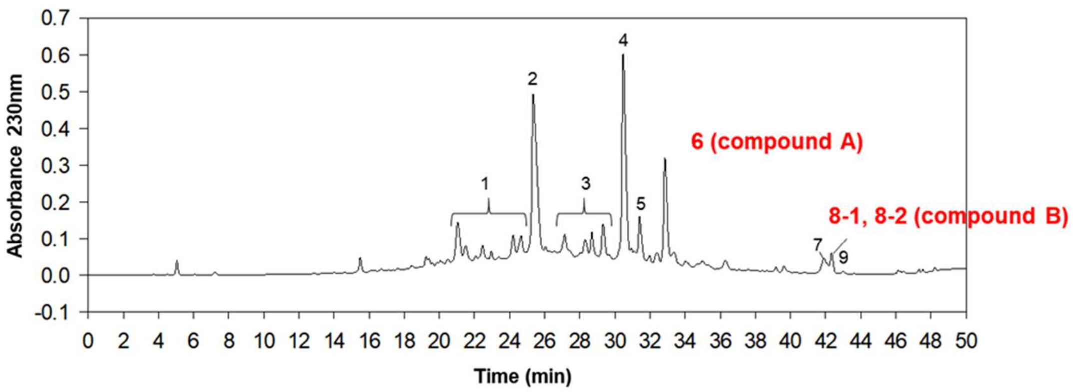

(b)

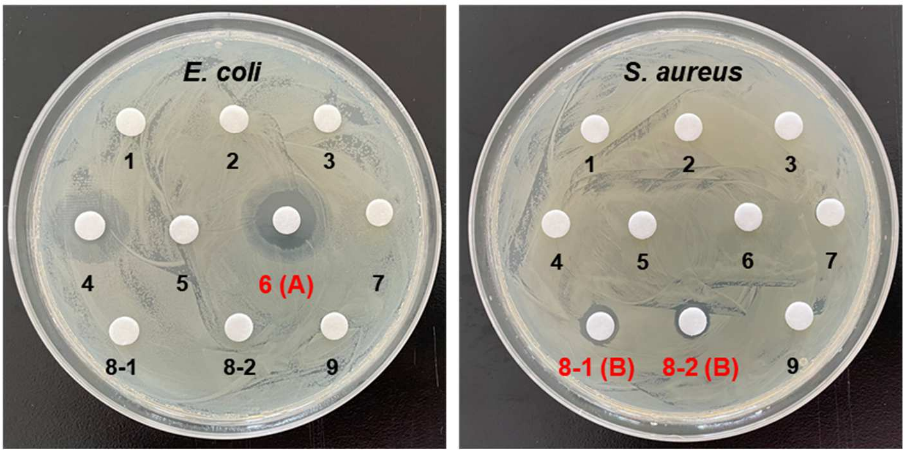

S2 Fig. The RP-HPLC chromatogram (a) and antibacterial activity (b) of the compounds in the 30% eluent of SPE purification are shown. Peaks 7 and 8 in the chromatogram correspond to compounds A and B, respectively.
