# Supplementary material for: New strain Brevibacillus laterosporus TSA31-5 produces both brevicidine and brevibacillin, exhibiting distinct antibacterial modes of action against Gram-negative and Gram-positive bacteria
Source: PLoS One. 2024 Apr 1;19(4):e0294474. doi: 10.1371/journal.pone.0294474 (PMC10984550; doi:10.1371/journal.pone.0294474)
Supplement: S3 Fig — The LC-MS analysis of compound A and B is depicted. RP-HPLC chromatograms (a and c) along with MS spectra (b and d) of the purified compounds A and B, respectively, are presented. (PDF) [file pone.0294474.s006.pdf]

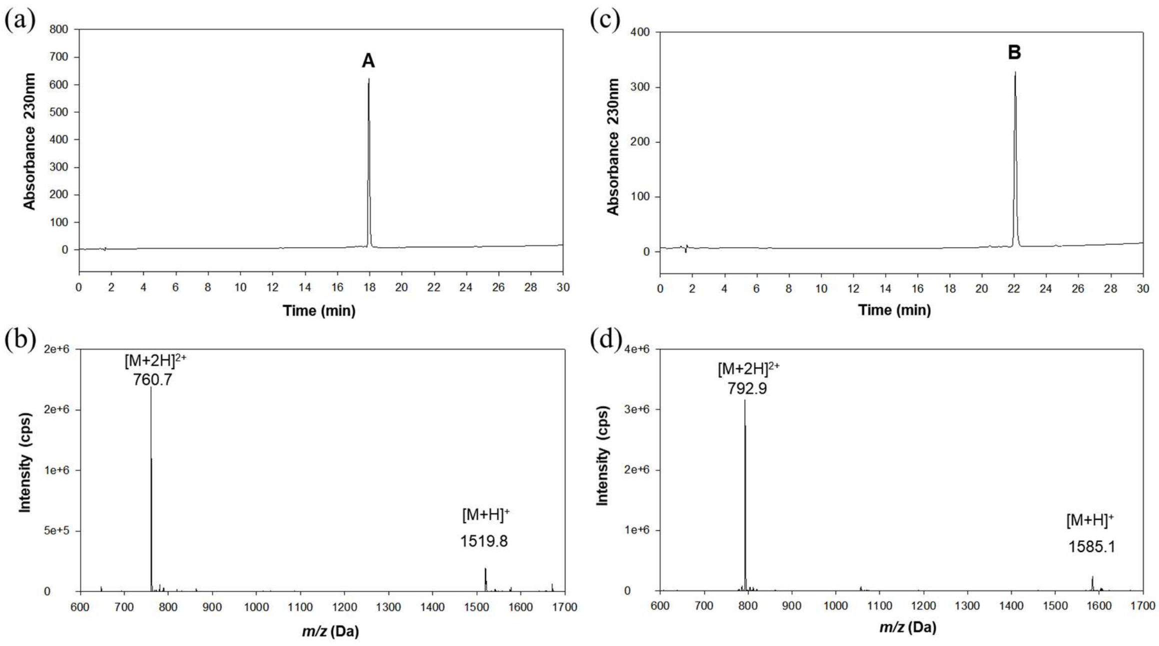

S3 Fig. The LC-MS analysis of compound A and B is depicted. RP-HPLC chromatograms (a and c) along with MS spectra (b and d) of the purified compounds A and B, respectively, are presented.
