# Supplementary figures and images for: New strain Brevibacillus laterosporus TSA31-5 produces both brevicidine and brevibacillin, exhibiting distinct antibacterial modes of action against Gram-negative and Gram-positive bacteria
Source: PLoS One. 2024 Apr 1;19(4):e0294474. doi: 10.1371/journal.pone.0294474 (PMC10984550; doi:10.1371/journal.pone.0294474)

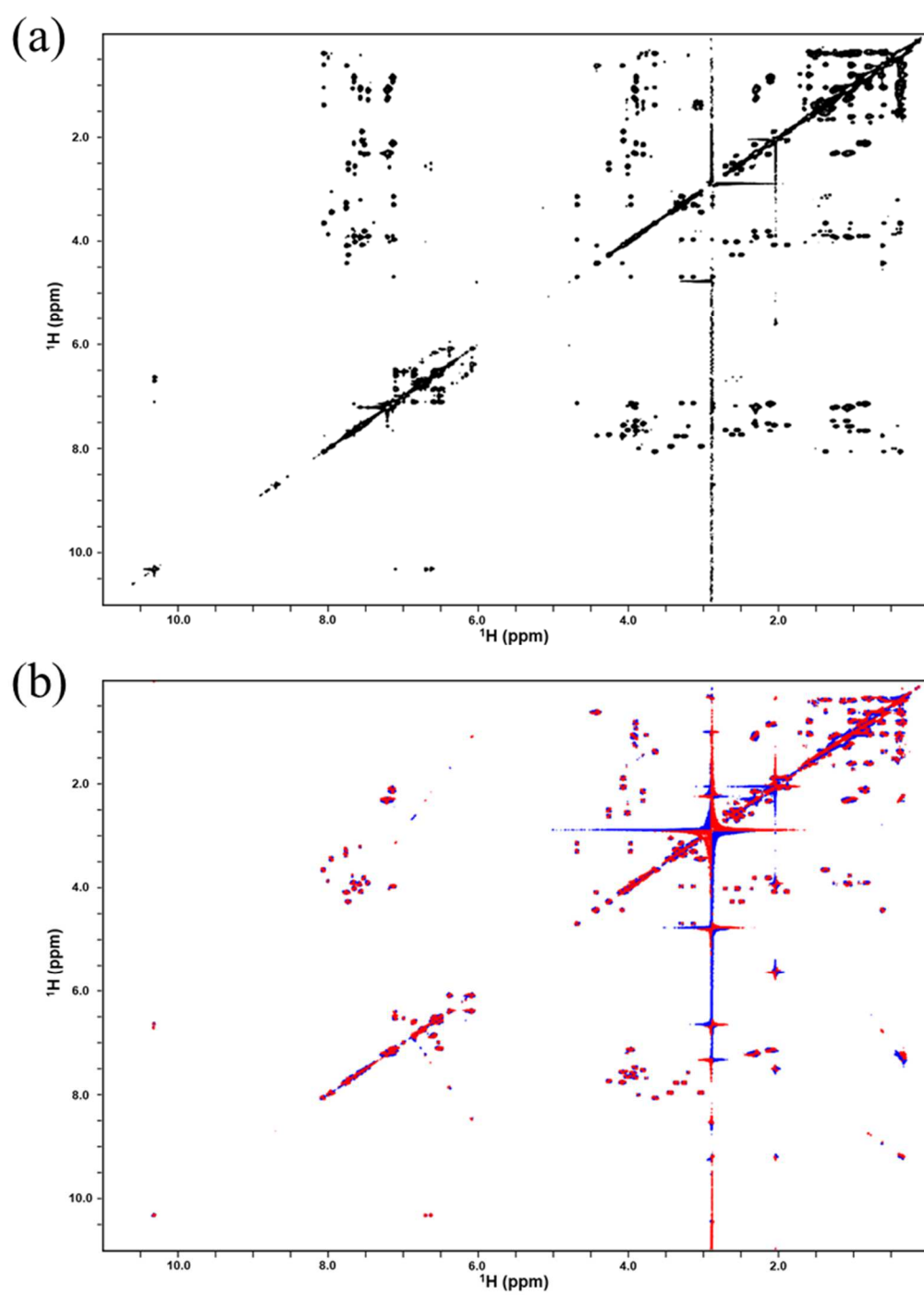

S4 Fig.  $^1\text{H}$  2D TOCSY (a) and DQF-COSY (b) NMR spectra of compound A.

Supplement: S4 Fig — 1H 2D TOCSY (a) and DQF-COSY (b) NMR spectra of compound A. (PDF) [file pone.0294474.s007.pdf]

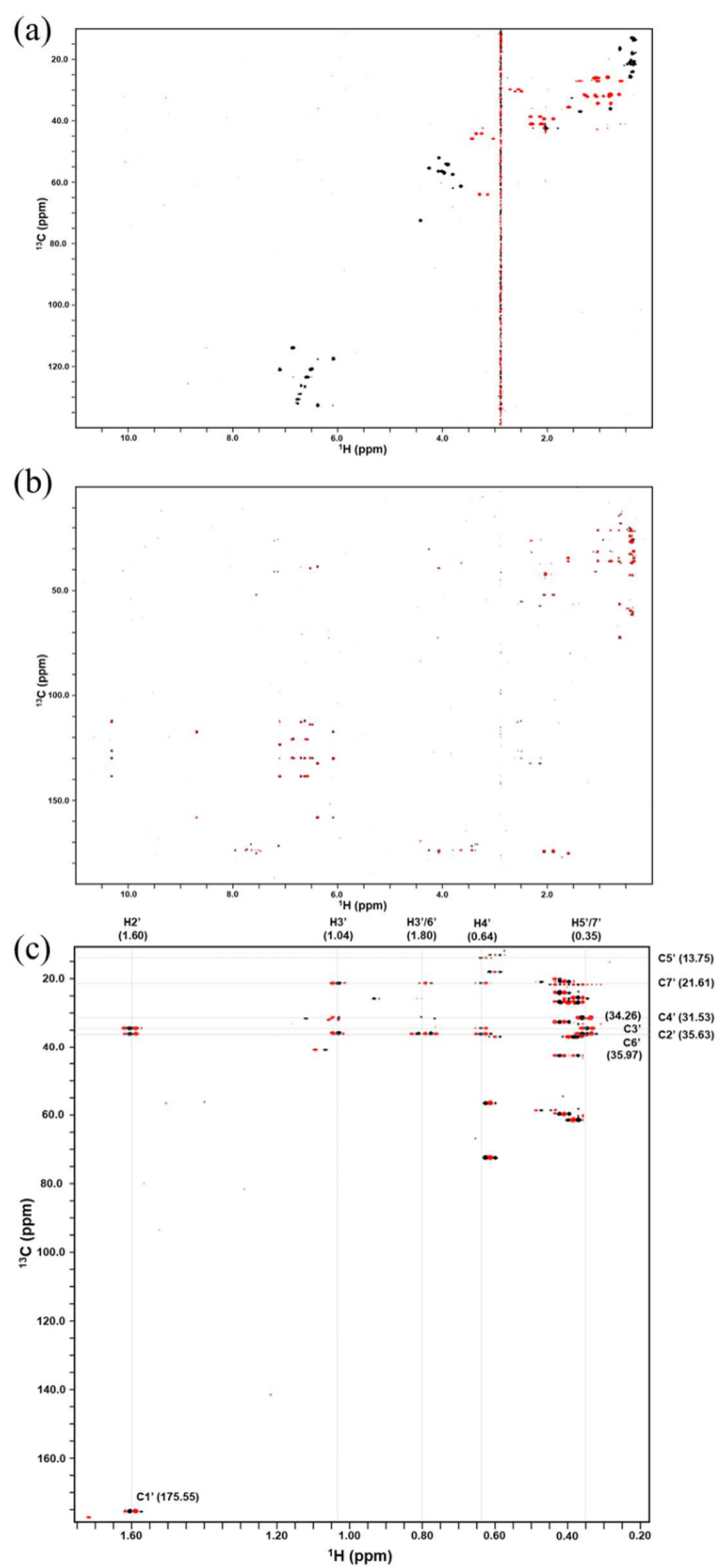

S5 Fig.  $^1\text{H}$ - $^{13}\text{C}$  HSQC (a), HMBC (b), and fatty acid chain region of HMBC (c) NMR spectrum of compound A

Supplement: S5 Fig — 1H-13C HSQC (a), HMBC (b), and fatty acid chain region of HMBC (c) NMR spectrum of compound A. (PDF) [file pone.0294474.s008.pdf]

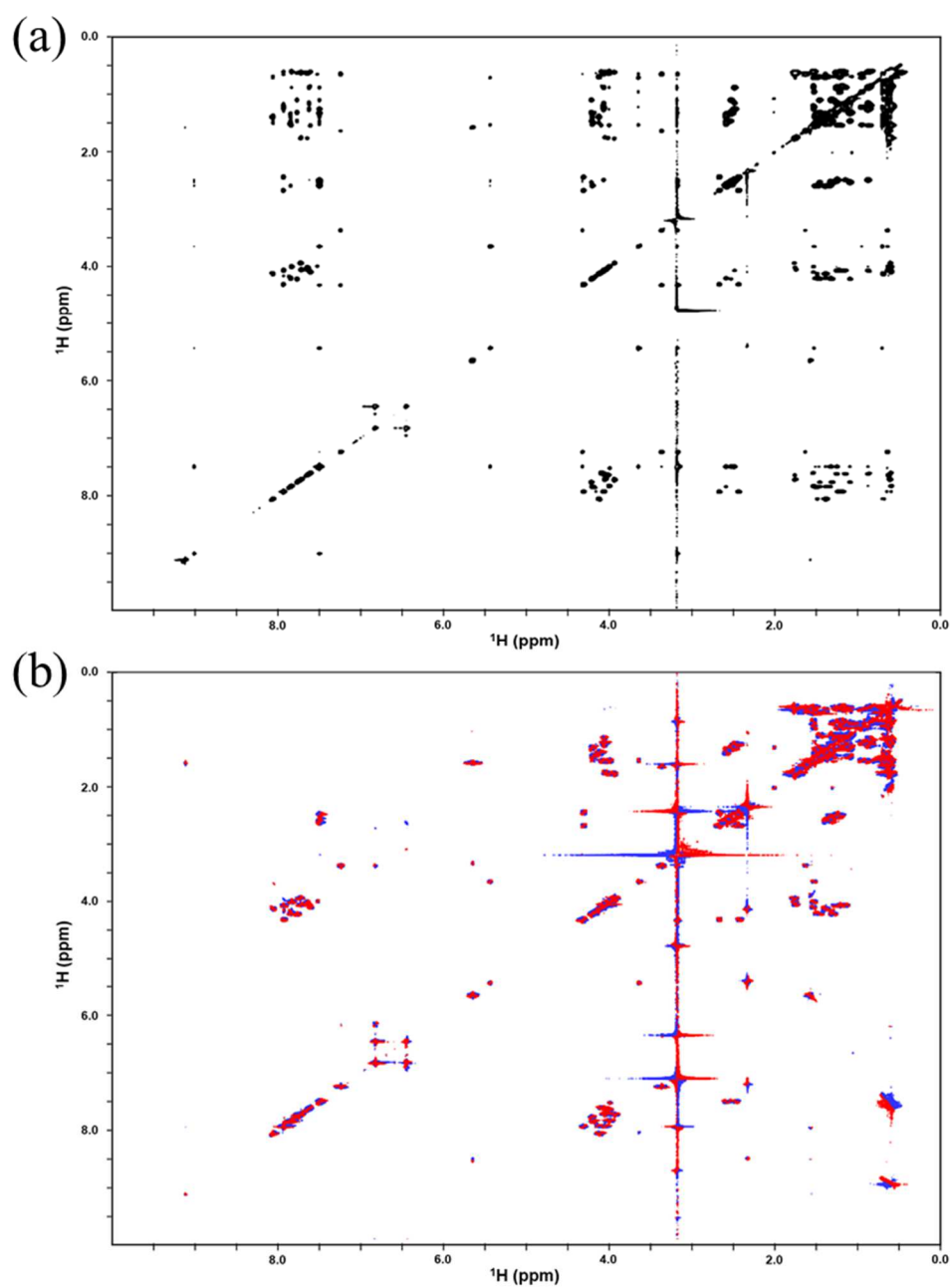

S6 Fig.  $^1\text{H}$  2D TOCSY (a) and DQF-COSY (b) NMR spectra of compound B

Supplement: S6 Fig — 1H 2D TOCSY (a) and DQF-COSY (b) NMR spectra of compound B. (PDF) [file pone.0294474.s009.pdf]

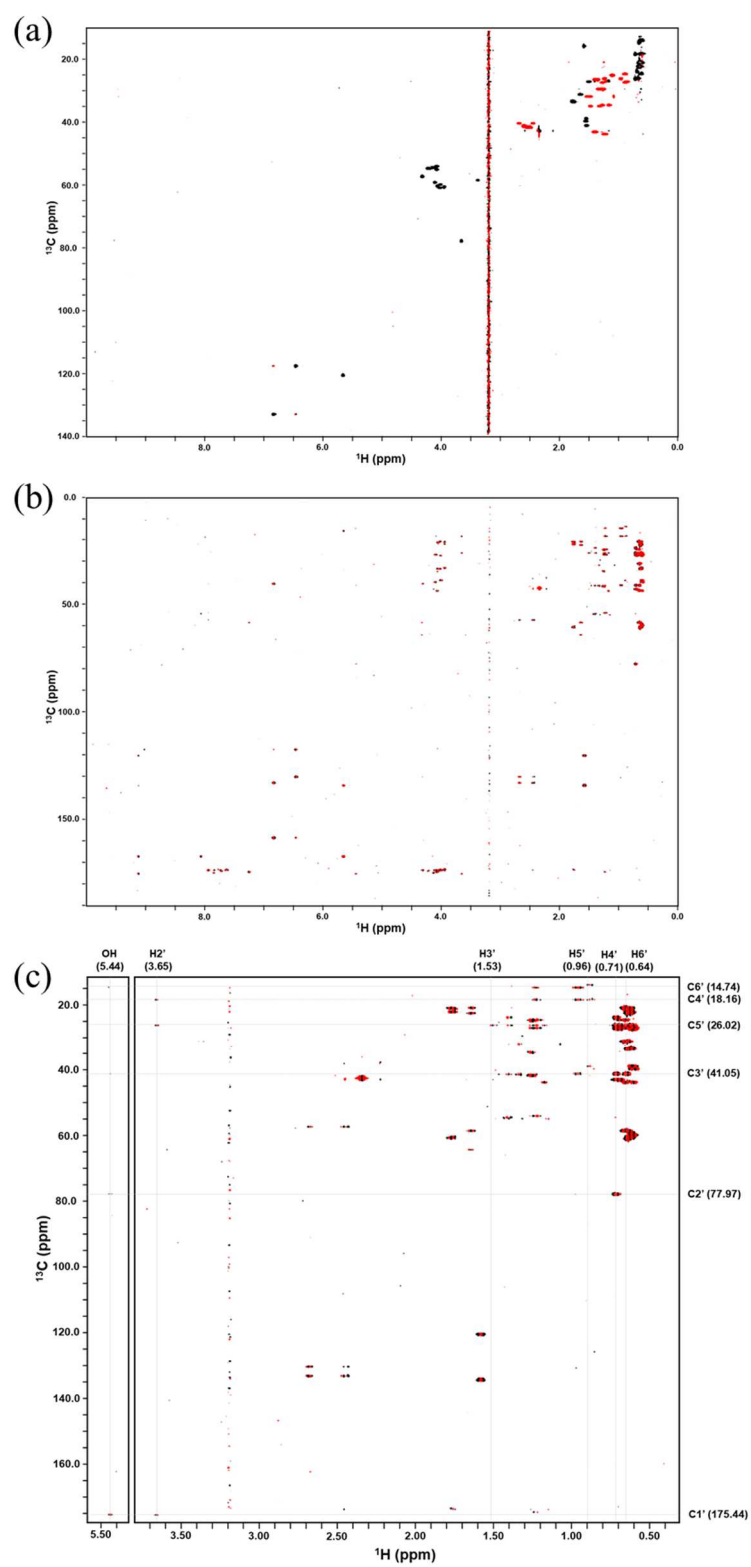

S7 Fig.  $^1\text{H}$ - $^{13}\text{C}$  HSQC (a), HMBC (b), and fatty acid chain region of HMBC (c) NMR spectrum of compound B

Supplement: S7 Fig — 1H-13C HSQC (a), HMBC (b), and fatty acid chain region of HMBC (c) NMR spectrum of compound B. (PDF) [file pone.0294474.s010.pdf]

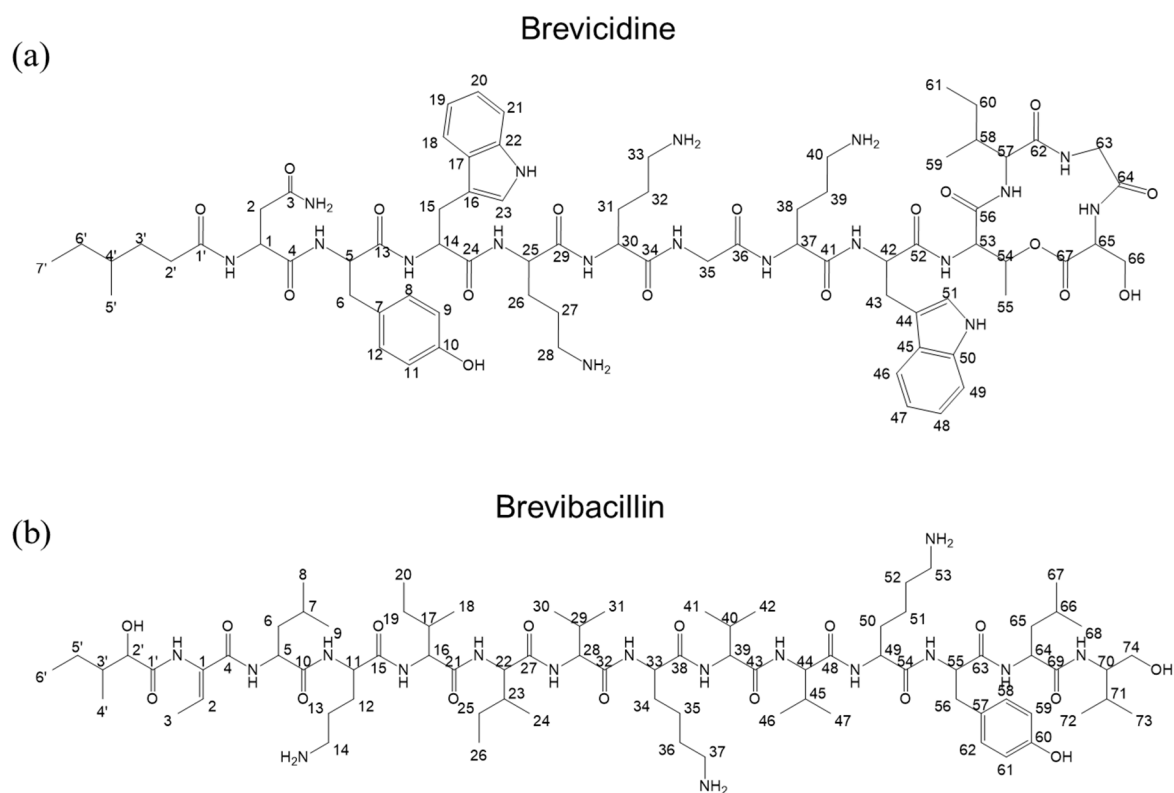

S8 Fig. Chemical structure of brevicidine (a) and brevibacillin (b)

Supplement: S8 Fig — Chemical structure of brevicidine (a) and brevibacillin (b). (PDF) [file pone.0294474.s011.pdf]

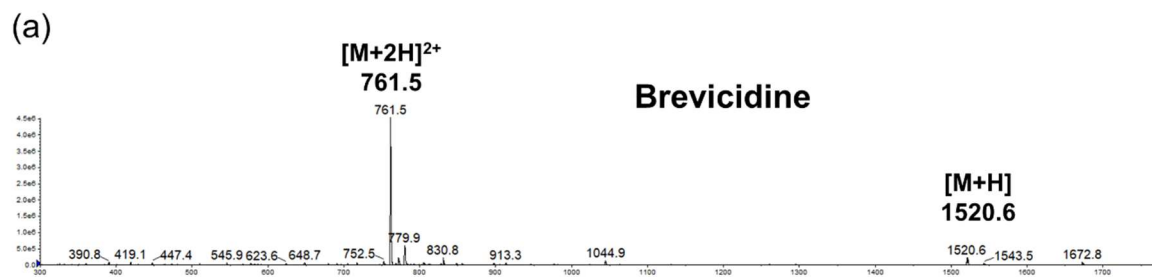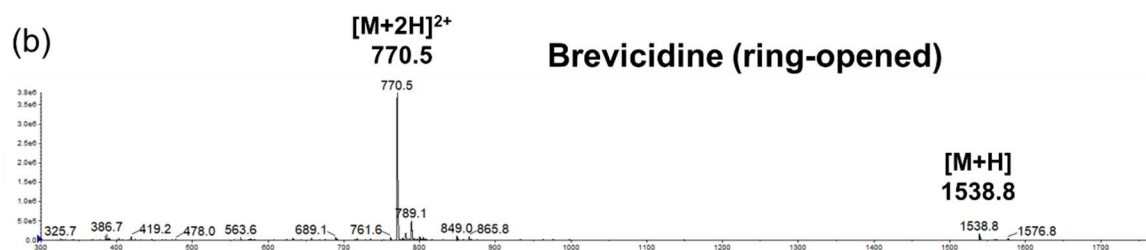

S9 Fig. LC-MS spectra of brevicidine (a) and ring-opened brevicidine (b) hydrolyzed at pH10.

Supplement: S9 Fig — LC-MS spectra of brevicidine (a) and ring-opened brevicidine (b) hydrolyzed at pH10. (PDF) [file pone.0294474.s012.pdf]
